# A novel approach to improving solubility of drugs: chelation-assisted solubility enhancement (CHASE)

**DOI:** 10.1080/14756366.2026.2704738

**Published:** 2026-07-27

**Authors:** TanPhat Nguyen, Nicola Bauer, Samer Gozem, Binghe Wang

**Affiliations:** Department of Chemistry and Center for Diagnostics and Therapeutics, Georgia State University, Atlanta, GA , USA

**Keywords:** Crown ether, chelation, solubility enhancement, cation–π interaction, partition coefficient

## Abstract

We explore a novel concept of metal Chelation-assisted Solubility Enhancement (CHASE). This study is based on complexation with Na^+^ or K^+^. A series of 6 compounds are studied by conjugating a hydrophobic moiety with benzo-15-crown-5. Log*P* values are determined in the absence and presence of Na^+^ or K^+^, respectively. The largest shift upon metal chelation is with compound **7** (a pyrenyl-crown ether conjugate), which has a log*P* of 3.61 in the absence of any metal ion and 0.99 and 1.45 in the presence of Na^+^ or K^+^, respectively. Such results clearly demonstrate the feasibility of the proposed approach. Beyond chelation, cation–π interactions seem to play a substantial role in determining the partitioning outcome for those with an aryl system. Such findings suggest the need for all future partition experiments to incorporate biologically relevant concentrations of metal ions. This feasibility study opens a new direction in improving drug solubility.

## Introduction

In the world of drug discovery and drug delivery, the issue of water solubility is a critical one^1–4^. This is true regardless of dosing route. Poor water solubility is said to reduce the performance of more than 10% of successfully marketed drugs^5^. This is because of the need for drug molecules to have at least some water solubility in order to be transported and to be efficacious. Further, drugs with solubility lower than their *K*_d_ values for the respective targets would have very little chance to be efficacious *in vivo.* In drug design, oil–water partitioning coefficient (log*P*) is often used as predictive parameter for measuring hydrophilicity in a biologically relevant manner^6^. A log*P* of 6 would only allow the concentration of the drug in aqueous solution to be no more than 1 nM in a partition experiment with a total concentration of 1 mM in the octanol phase. It should be noted that 1 mM is a very high number in a biological sense, considering physiological glucose concentration is below 7 mM. Compounds with a high log*P* tend to have very low absolute solubility in an aqueous solution, which negatively affects the chance to be developed further.

In overcoming issues of low water solubility and high oil–water partitioning coefficient, there are many existing approaches including the use of one or more co-solvents (i.e. propylene glycol and glycerol)^7^, micelles^8^^,^^9^, liposomes^10^, emulsions^11^, salt formation^12^, pH adjustment (for those drugs with solubility dependent on pH)^13^, and the use of inclusion complexation^14^, just to name a few^15–17^. Commonly used inclusion agents include calixarenes, cyclodextrin, cucurbit[n]urils, and phospholipids^14^^,^^17–22^. Another approach is the use of a prodrug strategy, which temporarily changes the physicochemical properties and delivery properties for a drug molecule and regenerates the original drug at the desired site and/or under the desired conditions^4^. In making prodrugs, the introduction of a polar and/or ionisable functional group (e.g. carboxyl, amino, and (poly)hydroxyl groups) is a commonly used method^23–25^. However, each of these functional groups carries its own potential problems depending on the context and circumstance.

**Figure 1.**
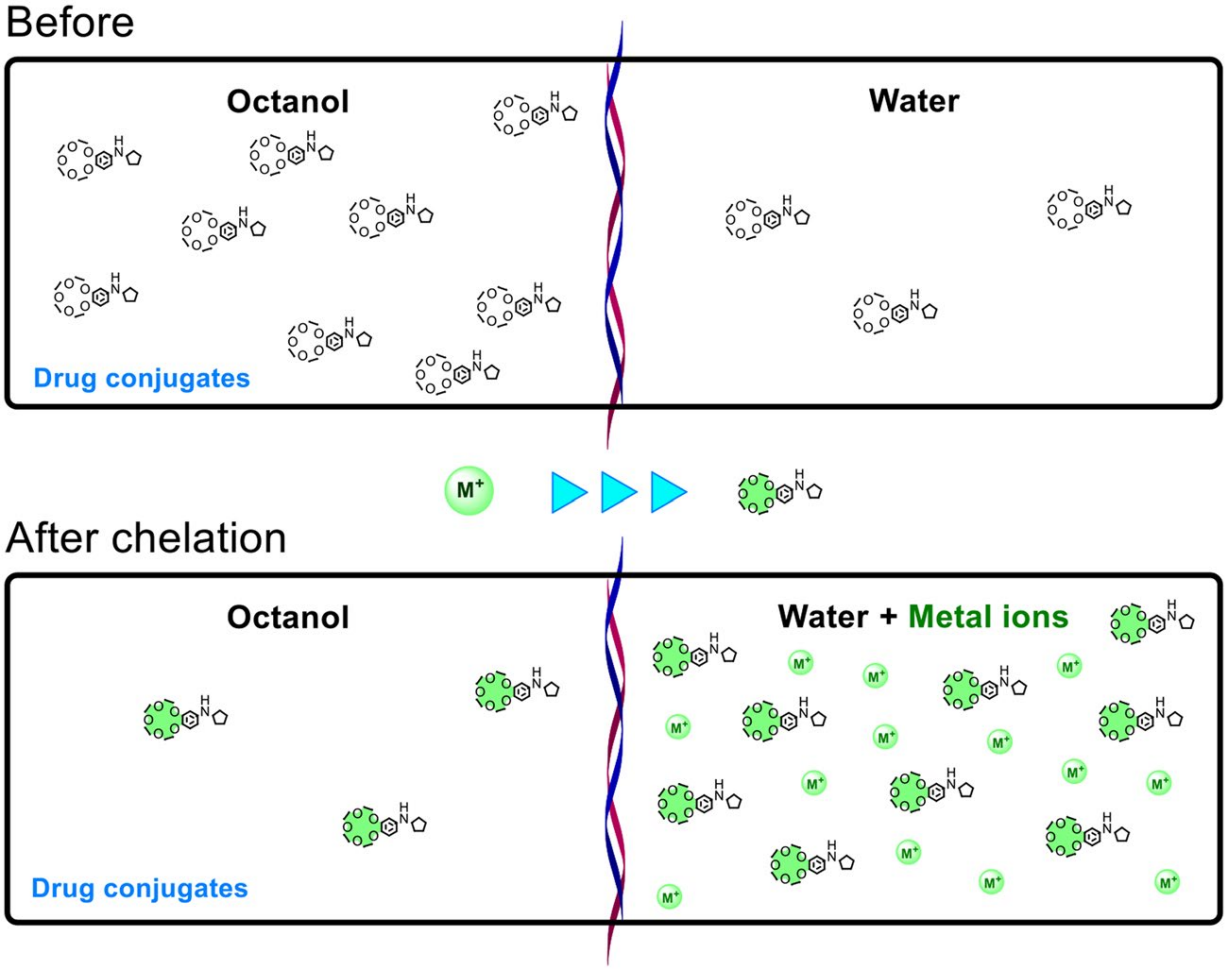
Concept of this work for solubility improvement based on metal chelation.

In this study, we aim to develop a new approach by exploring the possibility of using metal chelation as a way to improve solubility through the introduction of a chelator auxiliary group: chelation-assisted solubility enhancement (CHASE). This will pave the way for later use of reversible derivatization of a chelating moiety as a way for prodrug preparation. A key feature of chelation-assisted solubilisation is to take advantage of the natural presence of metal cations in the blood for solubilisation. Further, this approach does not involve the introduction of an ionisable group for solubility enhancement. Ionisable groups tend to introduce additional issues such as purification and the need for protection and deprotection chemistry during synthesis. One can envision the chelation-assisted solubilisation approach being complementary to the traditional approaches of introducing ionisable and/or polar groups. Figure 1 shows the concept of our work.

Our initial effort is focused on chelation with Na^+^ and K^+^ in model studies. Such design is predicated on the fact that human blood contains a high concentration of these two metal ions in the free form, with concentration being 135–145 mM of Na^+^ and 3.5–5.0 mM of K^+^^26^^,^^27^. It is important to note that Na^+^ concentration in the interstitial fluid (ISF) is similar to that of the blood concentration (95%–97%)^28^. There are other metal ions with high concentrations in the blood: 0.55–0.75 mM Mg^2+^ and 1.1–1.4 mM Ca^2+^^29^^,^^30^. Other metal ions in the blood have lower concentrations. Most other metal ions exist in bound forms except for Na^+^ and K^+^. For example, total Zn^2+^ concentration in the blood is about 10–18 μM with ca. 0.0001% in the free form^31^. Further, serum Fe^3+^/Fe^2+^ concentration is about 11–29 μM for females and 14–32 μM for males, with free iron being kept at virtually zero (ca. 10^–24^ M) to prevent tissue damage and to inhibit bacterial growth^32–35^. For this study, we aim to demonstrate the initial feasibility and understand the magnitude of solubility enhancement that one could expect by using very hydrophobic model structures.

## Results and discussion

### Design and synthesis

To study the feasibility of CHASE, we designed conjugates of a metal chelator with various hydrophobic moieties. For the metal chelators, there are choices of podands and crown ethers. We chose the latter in this feasibility study for various reasons including their simplicity and the large number of available crown ethers with known affinity for metal ions. Crown ethers are organic compounds known to bind metal ions in an aqueous environment^36^^,^^37^. Since the discovery by Charles J. Pedersen in 1967, the binding ability of these ligands with metal ions has been extensively studied. Among the ligands, 15-crown-5 exhibits strong affinity for sodium cation (Na^+^) due to its complementary cavity size for the formation of a 1:1 complex in both gas and solution phases [with log*K*_s_ at 25 °C being 3.25 (in methanol) and 0.79 (in water)]^38^^,^^39^. Its analog benzo-15-crown-5 (B15C5), however, is known to coordinate with both Na^+^ and K^+^. Interestingly, B15C5 prefers forming a sandwich (2:1) complex with K^+^ over forming (1:1) complex with Na^+^^40–45^. Hence, it can register as recognition moiety for binding K^+^ in aqueous solution^46^.

**Scheme 1.**
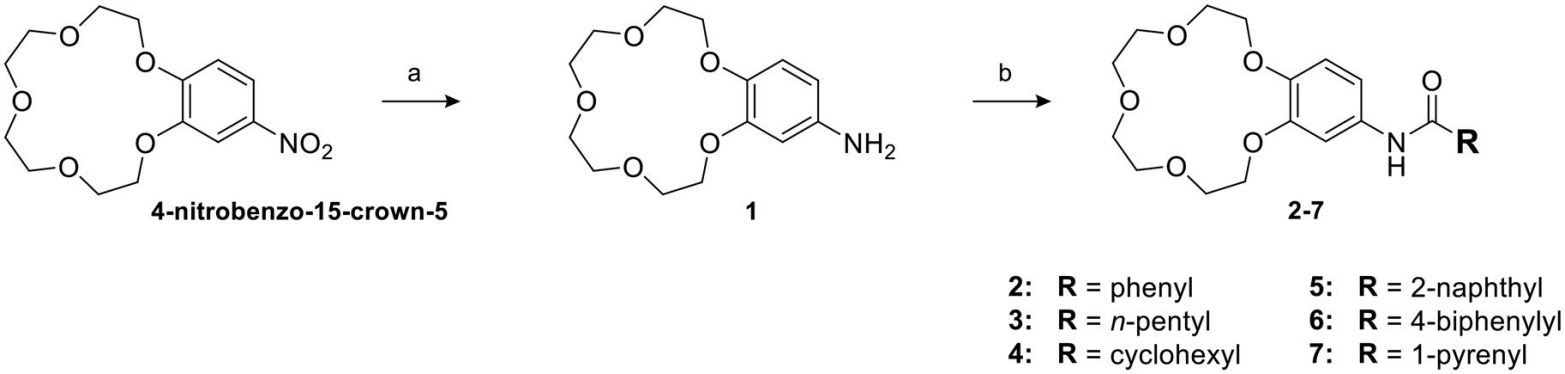
Synthesis of model crown ether analogs. (a) H_2_ balloon, Pd 10%/C, MeOH, r.t., 4 h, monitored by TLC until complete consumption of starting material; (b) either (i) R-COCl, Et_3_N, DCM, or (ii) R-COOH, EDC, DMAP, DCM, yield 30%–70%.

In designing a CHASE approach, we first selected a B15C5 moiety. We envision that, in the presence of cations at high concentration (e.g. Na^+^ in the blood serum and K^+^ in the cytosol), the B15C5 moiety can complex with the cation, leading to improved partition into the aqueous phase (Figure 1). To demonstrate proof of concept, we are interested in the synthesis and assessment of partitioning coefficients of conjugates of B15C5 with various hydrophobic structures such as phenyl, biphenyl, naphthyl, cyclohexyl, pentyl, and pyrenyl groups representing aromatic, polyaromatic, and alkyl groups. These moieties with different lipophilicity are chosen from previously reported data (Table 1)^47^. The synthesis is shown in Scheme 1. In brief, 4-nitrobenzo-15-crown-5 was reduced to the corresponding 4-aminobenzo-15-crown-5 (**1**) with 100% conversion (TLC)^48^ before subsequent amidation, leading to the desired products **2**–**7** in 30%–70% yield.

**Table 1.** Experimentally determined log*P* values of compounds **2**–**7**.

Compound	Literature log*P^47^*	Experimental log*P*		
		Oct/H_2_O	Oct/K^+^ 144 mM	Oct/Na^+^ 144 mM
**2**	benzene: 2.13	1.62 ± 0.01	1.35 ± 0.01	1.39 ± 0.01
**3**	*n*-pentane: 3.39	2.30 ± 0.01	1.70 ± 0.01	1.75 ± 0.01
**4**	cyclohexane: 3.44	2.30 ± 0.01	1.67 ± 0.01	1.70 ± 0.01
**5**	naphthalene: 3.59	2.72 ± 0.01	1.43 ± 0.01	1.87 ± 0.02
**6**	biphenyl: 3.95	3.25 ± 0.01	2.85 ± 0.04	2.91 ± 0.03
**7**	pyrene: 4.88	3.61 ± 0.01	0.99 ± 0.01	1.45 ± 0.01

Next, we examined how the presence of K^+^ or Na^+^ influences each compound’s partitioning coefficient (log*P*) and the magnitude of the differences when compared with the log*P* in the absence of any metal ions. Specifically, for assessing their partition coefficients, we conducted three types of experiments: (1) between octanol/water, (2) between octanol/aqueous Na^+^ solution (144 mM), and (3) between octanol/aqueous K^+^ solution (144 mM). The first set of experiments assesses the intrinsic effect of the crown ether on the compound’s partition properties. The second set of experiments simulates the scenario in the blood with high Na^+^ concentration (135–145 mM)^26^^,^^27^. The third set of experiments simulates the intracellular environment because of the known high intracellular concentration of K^+^ (140–150 mM)^26^.

### Determination of partition coefficients between *n*-octanol and pure water

**Figure 2.**
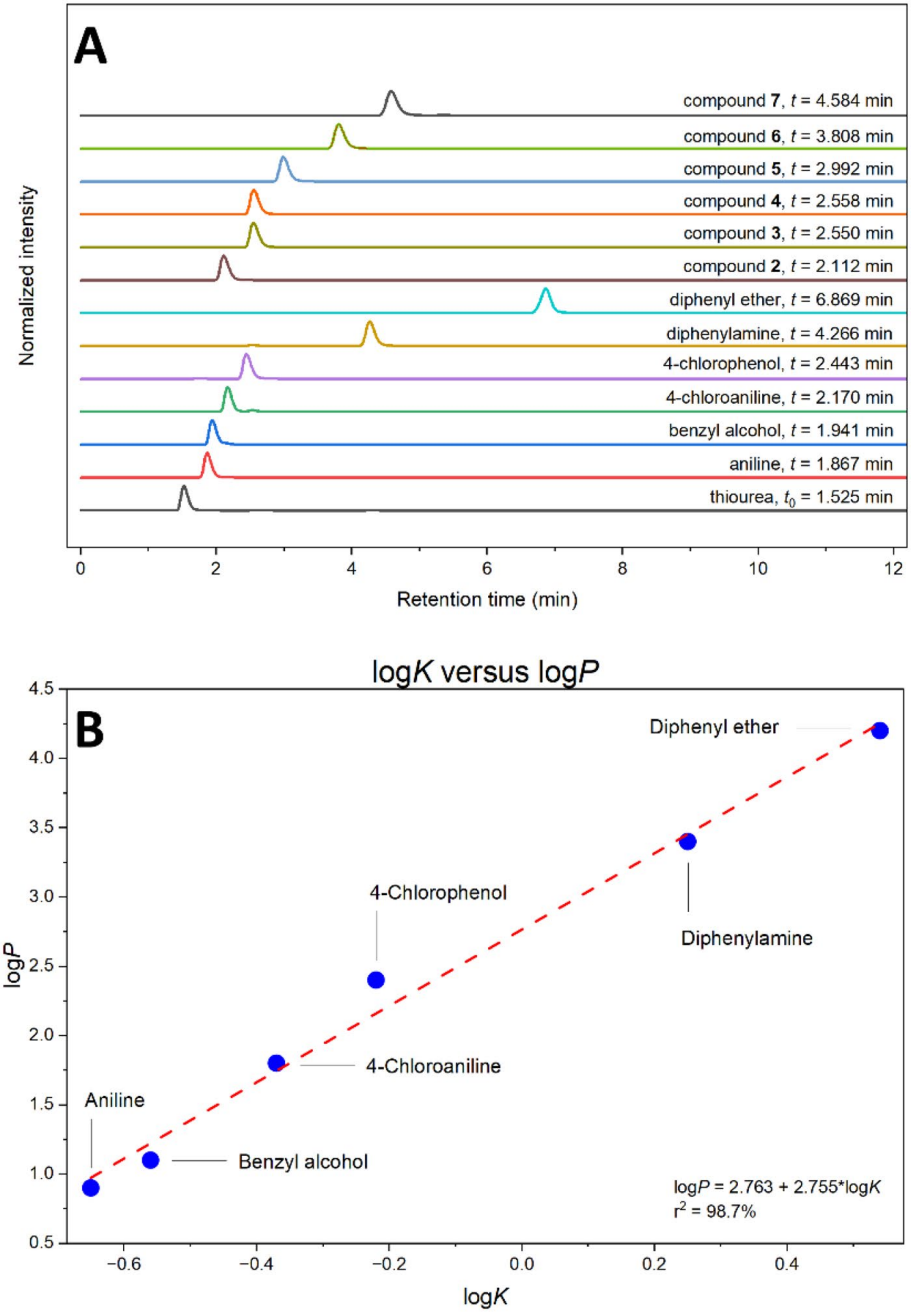
(A) Representative HPLC chromatograms showing retention times of seven references and investigated compounds (monitored at UV_210 nm_). (B) Calibration curve showing a linear relationship between experimental log*K* and known log*P* values.

log*P* estimation was done according to OECD Guide No. 117 (HPLC method for log*P* estimation in the range of 0–6)^49^^,^^50^. Specifically, seven reference compounds with known log*P* values from a reference list were used to measure their retention times *t* on HPLC column^49^. Solvents to prepare analyte samples and mobile phase were identical: MeOH:H_2_O (3:1, v/v). Since thiourea is minimally retained in HPLC, its retention time is defined as the dead time *t*_0_. Based on retention times (*t* and *t*_0_), each compound’s capacity factor, *K*, is calculated (See SI for details). The log*K* values are then used to build a calibration curve with the known log*P* values for determining partition coefficients of investigated compounds. Figure 2(A) shows representative HPLC chromatograms of the standards (aniline, benzyl alcohol, 4-chloroaniniline, 4-chlorophenol, diphenylamine, and diphenyl ether). The calculated log*K* values are shown in Table S1. Using the calibration curve (Figure S1), the corresponding log*P* values were calculated and listed in Table 1.

Here, we need to note that we did not use the traditional shake-flask method because of the high log*P* for some compounds and low UV extinction coefficients, which gave us sensitivity issues with the compounds studied.

### Determination of partition coefficients between *n*-octanol and solutions of cations

For this determination, we decided to use the traditional shake-flask method based on OECD Guide No. 107 (shake-flask method) for log*P* estimation in the presence of metal ions^47^^,^^51–53^. We did not use the HPLC method because of the need to use high concentrations of metal cations (>140 mM), which tends to cause HPLC clogging problems. Prior to partitioning, two phases were mutually saturated at room temperature for at least 24 h. Then, test compounds were partitioned between two phases, with one phase being *n*-octanol and the other phase being either K^+^ 144 mM or Na^+^ 144 mM solution, overnight. The concentration of the analyte in each phase was determined using standard curves which were built by HPLC and UV-Vis analyses. This approach allowed determination of new partition coefficients between octanol and solution of K^+^ or Na^+^ (see the supplementary material for experimental details and the standard curves, Figures S4–S12).

Because we used two different methods for log*P* estimations of these compounds in the presence and absence of metal ions, we measured the log*P* of **2** in the absence of metal ions using both the HPLC and shake-flask methods to make sure that valid comparison can be made. We used **2** for this purpose because of its low log*P*, which is conducive to log*P* determination using both methods. Data are presented in Table S5. With the consistency between the log*P* value using the shake-flask method (1.58) and the HPLC method (1.62), we feel confident in comparing data from these two methods^49^. All data are collectively listed in Table 1.

From the data, several lines of findings are apparent. First, attaching a crown ether to a hydrophobic moiety generally helps improve partitioning into the aqueous phase, as one would expect. Such improvement is smaller with the less hydrophobic moiety. For example, the log*P* is 2.13 for benzene^47^ and 1.62 for the conjugate (compound **2**). This is a difference of 0.51. In contrast, the log*P* is 4.88 for pyrene^47^ and 3.61 for the conjugate (compound **7**), with a difference of 1.27. Such findings are understandable since the crown ether moiety has multiple heteroatoms and is known to have good water solubility^45^^,^^54^^,^^55^. Furthermore, we would expect to see more meaningful log*P* improvements with the more hydrophobic compounds such as compound **7**. Second, in the presence of a metal ion, a substantial decrease in the log*P* value was observed. For example, the log*P* value was determined to be 1.62 for conjugate **2** in the absence of a metal ion, 1.39 in the presence of Na^+^, and 1.35 in the presence of K^+^. Again, the solubilisation effect of metal chelation is the greatest with the most hydrophobic unit, pyrene. Specifically, the log*P* value was found to be 4.88 for pyrene^47^, 3.61 for conjugate **7** in the absence of a metal ion, 1.44 in the presence of Na^+^, and 0.99 in the presence of K^+^. Third, the difference between the effects of K^+^ and Na^+^ seems to be small in most cases. This is understandable. Although B15C5 is known to have a preference for K^+^, with log*β*_i_ (logarithm of stability constant) being approximately 4.80 ± 0.05 (i = 2) for K^+^ and 2.26 ± 0.05 (i = 1) for Na^+^^56^, the concentrations of the salts used well exceeded what was needed to saturate binding. As a result, one would not expect to see much difference when K^+^ and Na^+^ were used at such high concentrations. Fourth, large differences between the log*P* in the absence and presence of a metal ion (K^+^ and Na^+^) are a general phenomenon observed with all the analogs. Fifth, there seems to be some additional force(s) at play influencing the partitioning of these compounds. For example, the log*P* value was found to be 2.72 for compound **5** in the absence of a cation, 1.87 in the presence of Na^+^, and 1.43 in the presence of K^+^. Similarly, the log*P* value was found to be 3.61 for compound **7** in the absence of a cation, 1.45 in the presence of Na^+^, and 0.99 in the presence of K^+^. These represent substantial changes in partitioning coefficient. On the other hand, the log*P* value was found to be 3.25 for compound **6** in the absence of a cation, 2.91 in the presence of Na^+^, and 2.85 in the presence of K^+^. The effect of metal chelation seems to be much smaller in the case of compound **6** than that of compound **5** or **7**.

In considering the possible reasons for the differential effects of metal ions on the log*P* of the compounds studied, we turned to cation–π interactions, which are well known and considered strong enough that their interaction energy may exceed that of hydrogen bonding^57^. Further, cation–π interactions are much stronger in cases with extended conjugation such as napthalene^57–60^. Conversely, cation–π interactions substantially modify the electronic properties of the π system to the point that it could fundamentally change the π system and even alter the chemical reactivity^61^. Furthermore, conjugating a crown ether with a metal-chelator is known to create synergy in binding^62^.

To compare the strength of cation–π interactions between the aromatic substituents (benzene, biphenyl, naphthalene, and pyrene) and Na^+^ and K^+^, we carried out density functional theory (DFT) calculations on model systems. The geometries of the aromatic compounds were prepared in isolation and in the presence of the Na^+^ or K^+^ ions, which at first were manually placed above the plane of the aromatic rings at ∼3 Å. Those preliminary structures served as starting points for geometry optimizations carried out using the ωB97X-D DFT functional^63^ and aug-cc-pVDZ basis set^64^. The all-electron aug-cc-pVDZ-X2C basis set was used for K^+^^65^. The choice of method was motivated by the inclusion of built-in empirical dispersion correction in the functional and diffuse functionals in the basis set, both of which are important for properly capturing the cation–π interactions. The use of diffuse functions on the basis set also mitigates issues arising from basis set superposition and incompleteness errors^66^^,^^67^. For pyrene, the optimizations were started from three different geometries, one where the cations were centred above the pyrene centre of mass, one displaced off-centre along the long axis, and one centred off-centre along the shorter axis. The geometry yielding the lowest energy was selected for further energy analysis.

Calculations were carried out in the gas phase and then repeated using a polarisable continuum solvation model to include the effect of a water solvent through the integral equation formalism (IEF-PCM) with a dielectric constant of 78.35^68^.

The cation–π interaction energy was calculated as the difference in energy between the interacting aromatic-cation complex and the energies of the isolated aromatic molecules and Na^+^/K^+^.

**Figure 3.**
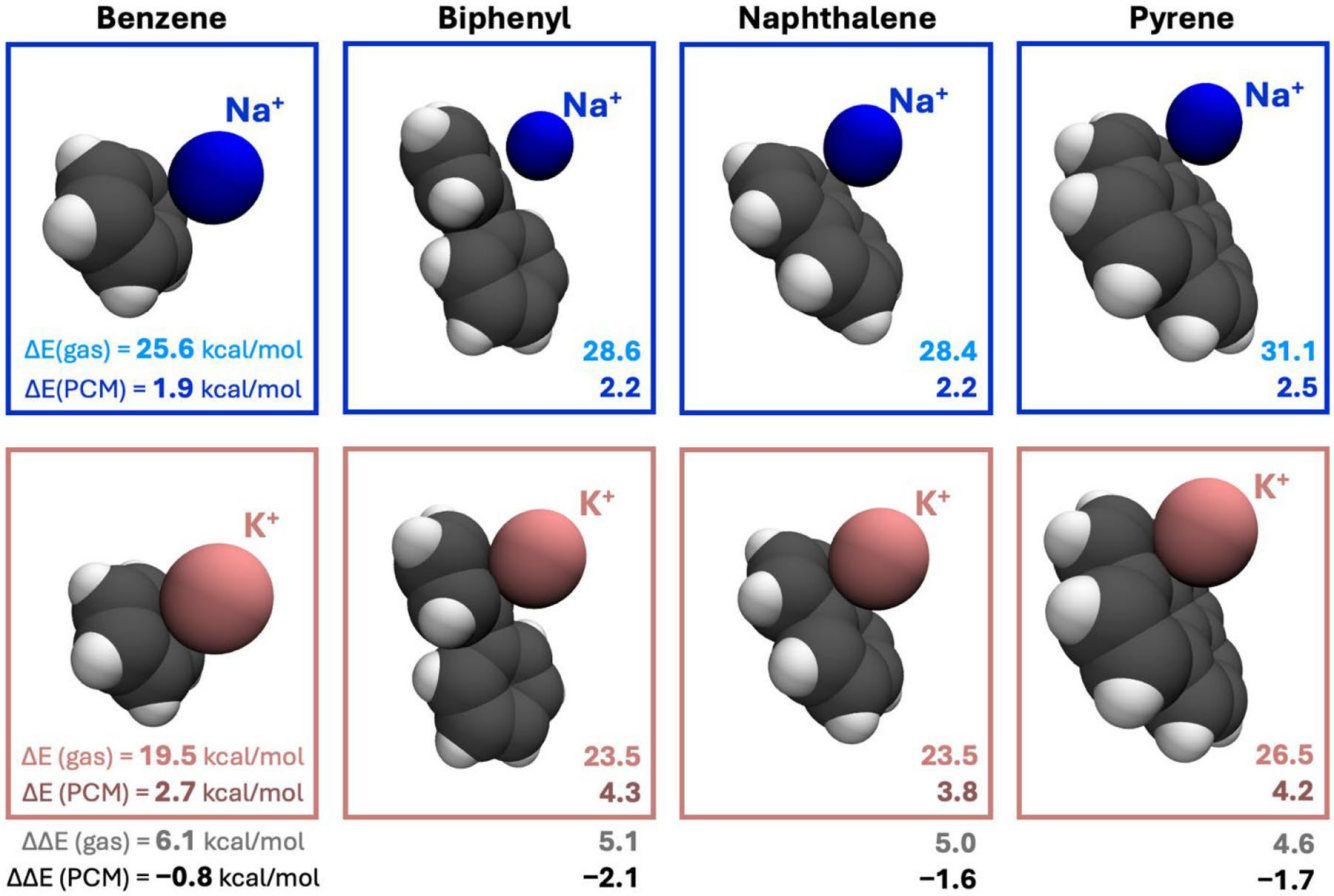
Cation–π interaction geometries and energies (in kcal/mol) obtained from density functional theory (DFT). The atoms are represented using their van der Waals radii. The top row shows aromatic–Na^+^ interactions, while the row below it shows aromatic–K^+^ interactions. The computed cation–π interaction energies are shown for the gas phase (lighter color) and for the PCM (darker color) calculations. The difference in energy (ΔΔE) at the bottom reflects the difference in affinity of each aromatic compound between Na^+^ and K^+^ ions for the gas phase (grey) and in PCM (black). Note that the binding energies are reported here as absolute values (the negative sign sometimes used by convention is dropped).

Figure 3 shows the cation–π interaction energies computed from DFT for different aromatic–ion pairs. The gas phase calculations show that the strength of the cation–π interaction between all aromatic compounds is stronger for Na^+^ compared to K^+^ (i.e. all ΔΔE values are positive). This is consistent with calculations previously reported in the literature^57^^,^^69^. The stronger binding could be understood through the higher charge density on Na^+^, which interacts more strongly with the aromatic π orbital electrons than K^+^. However, the ΔΔE becomes negative in all cases when the effect of the water solvent is included through PCM. The higher charge concentration of Na^+^ also means that it is better solvated when it dissociates from the aromatic compound compared to K^+^. Therefore, in solution, the cation–π interaction becomes stronger for K^+^ compared to Na^+^. This is consistent with the experimental results in Table 1 that consistently show a lower log*P* for Oct/K^+^ compared to Oct/Na^+^.

Figure 3 predicts a stronger binding energy to both cations with increase in size of the aromatic system, consistent with expectations that the increase in the polarizability of the aromatic system would increase the strength of the cation–π interaction^70^, but with a stronger affinity to K^+^. With the exception of biphenyl, the calculated trends in ΔΔE in PCM are highly consistent with the log*P* values reported in Table 1.

To the best of our knowledge, we have found no reports on cation–π interactions with biphenyl. Its increased flexibility, leading to it adopting a twisted conformation (see Figure 3), may disrupt cation–π interactions in room temperature conditions where the two linked benzene rings are free to rotate. Indeed, repeating the cation–π interaction calculation while constraining the two rings to be coplanar does reduce the energy of the interaction to 2.2 kcal/mol for Na^+^ and 3.9 for K^+^ in PCM, lowering the ΔΔE to −1.7 kcal/mol instead of −2.1. Other factors that may contribute to the out-of-trend behaviour of biphenyl compared to the other rigid aromatic rings remain a topic for future exploration.

Based on literature studies of cation–π interactions as well as our own computational work with these specific compounds, it is reasonable to expect cation–π interaction to be a force in affecting the log*P* value. There may be other intricate and interesting details related to biphenyl, such as its twisted relationship between the two phenyl rings and the effect of cation–π interaction on the other phenyl ring^71–73^. Of course, to capture such subtle differences would require more sophisticated models that can accurately balance solvation energies with cation–π interactions, incorporate the full model compound (i.e. including the crown ether), and/or account for effects such as sandwich (2:1) coordination of ions. Such issues may merit further studies by the theoretical community, and we plan to conduct further computational work.

## Conclusions

In summary, conjugation of a chelator moiety can be used for CHASE. This solubility enhancing effect is more than the added polarity of a chelator. Through this limited study, we can draw several conclusions. *First*, complexation with metal ions seems to offer a chance for substantial improvement in water solubility. *Second*, exploring the concentration difference of metal ions, especially Na^+^ and K^+^, between the intracellular and extracellular space can be an interesting way of achieving intracellular enrichment. *Third*, in addition to simple polarity change upon chelation, cation–π interactions seem to play a substantial role in influencing the log*P* values of the compounds examined. *Fourth*, our results strongly suggest the need for all future partitioning experiments to be conducted in the presence of physiologically relevant cations at an appropriate concentration: e.g. 1X PBS instead of water. This is literally the well-known log*D* value, except this concept of log*D* is mostly commonly discussed in the context of compounds with ionisable functional groups, while the significant effect of cation–π interactions is commonly overlooked. *Fifth*, the difference between log*P-*Na^+^ and log*P-*K^+^ will offer significant insights into intracellular partitioning propensity. Therefore, there is much more work to do in understanding the effect of cation–π interactions on solubility enhancement and partitioning among compartments. The impact can be very significant. *Sixth*, there are several directions for future computational modelling work to better understand the energy contributions to cation–π interactions in solution and to improve the level of theory^74^. We hope and expect this study to stimulate broad discussions and extensive additional experimental and computational work.

## Experimental section

### General methods

All commercial chemicals and anhydrous solvents were of reagent grade and used without further purification. Column chromatography was carried out using flash silica gel (230–400 mesh) from Sigma-Aldrich. TLC analysis was conducted on silica gel plates (Sorbent Silica G UV254). ^1^H– (400 MHz) and ^13^C–NMR (101 MHz) spectra were recorded on Bruker Avance 400 MHz spectrometer. Chemical shifts (δ values) and coupling constants (*J* values) are given in ppm and hertz, respectively, using the respective solvent as internal reference. Mass spectrometric analyses (HR-ESI-MS) were performed by the Mass Spectrometry Facilities at Georgia State University.

### General procedure for preparation of 2,3,5,6,8,9,11,12-octahydrobenzo[*b*][1,4,7,10,13]pentaoxacyclopentadecin-15-amine (1)

To a 50 ml flask, 4-nitrobenzo-15-crown-5 (0.6956 g, 1 eq.) and Pd 10%/C (0.05 eq.) were added together with 5 ml MeOH. The mixture was stirred under H_2_ atmosphere for 4 h. After that, it was filtered through Celite and the solvent from the filtrate was removed under reduced pressure. The amine product **1** is a known compound^48^ and was used in the next step without purification.

### General procedure for preparation of compounds 2–5

To a 0.1 M solution of **1** in anhydrous, DCM were added the corresponding acyl chloride (1.25 eq.) and triethylamine (TEA, 3 eq.). The reaction mixture was stirred at ambient temperature overnight, followed by work-up with HCl 1 M solution and brine, and extraction with DCM. The combined organic phase was dried over Na_2_SO_4_. After solvent removal under reduced pressure, the final product was purified using flash chromatography over silica gel using MeOH/DCM as the eluent.

### General procedure for preparation of compounds 6–7

To a 0.1 M solution of carboxylic acid in anhydrous, DCM were successively added 1-ethyl-3-(3-dimethylaminopropyl)carbodiimide (EDC, 1.2 eq.), 4-dimethylaminopyridine (DMAP, 0.25 eq.), and the amine **1** (1.2 eq.). The resulting solution was stirred at ambient temperature overnight, followed by work-up with HCl 1 M solution and brine, and extraction with DCM. The combined organic phase was dried over Na_2_SO_4_. After solvent removal under reduced pressure, final product was purified by flash-chromatography over silica gel using MeOH/DCM as the eluent.

### *N*-(2,3,5,6,8,9,11,12-octahydrobenzo[*b*][1,4,7,10,13]pentaoxacyclopentadecin-15-yl)-benzamide (2)

Yield: 34%. δ_H_: 8.33 (s, NH), 7.84 (d, *J* = 7.1 Hz, 2H), 7.51–7.34 (m, 4H), 7.01 (dd, *J* = 8.6, 2.4 Hz, 1H), 6.75 (d, *J* = 8.6 Hz, 1H), 4.10–4.01 (m, 4H), 3.89–3.83 (m, 2H), 3.83–3.78 (m, 2H), 3.74–3.68 (m, 8H). δ_C_: 165.9, 149.2, 145.8, 135.0, 132.4, 131.7, 128.7, 127.2, 114.7, 113.1, 107.4, 71.0, 70.9, 70.5, 70.4, 69.6, 69.5, 69.4, 68.7. HRMS (ESI, positive ion mode): calc. for C_21_H_26_NO_6_ 388.1760 ([M + H]^+^), found 388.1743. HRMS (ESI, negative ion mode): calc. for C_21_H_24_NO_6_ 386.1604 ([M–H]^–^), found 386.1607.

### *N*-(2,3,5,6,8,9,11,12-octahydrobenzo[*b*][1,4,7,10,13]pentaoxacyclopentadecin-15-yl)hexamide (3)

Yield: 72%. δ_H_: 9.79 (s, 1H), 7.33 (d, *J* = 1.3 Hz, 1H), 7.07 (dd, *J* = 8.6, 1.2 Hz, 1H), 6.87 (d, *J* = 8.6 Hz, 1H), 4.00 (s, 4H), 3.83–3.71 (m, 4H), 3.60 (s, 8H), 3.04 (q, *J* = 7.1 Hz, 2H), 2.25 (t, *J* = 7.4 Hz, 2H), 1.63–1.51 (m, 2H), 1.28–1.13 (m, 5H). δ_C_: 170.8, 148.3, 144.1, 133.5, 114.5, 111.4, 106.0, 70.2, 70.1, 69.7, 69.6, 68.9, 68.8, 68.7, 68.2, 36.3, 30.9, 24.8, 21.9, 13.9. HRMS (ESI, positive ion mode): calc. for C_20_H_32_NO_6_ 382.2225 ([M + H]^+^), found 382.2242. HRMS (ESI, negative ion mode): calc. for C_20_H_28_NO_6_ 380.2078 ([M–H]^–^), found 380.2089.

### *N*-(2,3,5,6,8,9,11,12-octahydrobenzo[*b*][1,4,7,10,13]pentaoxacyclopentadecin-15-yl)cyclohexanecarboxamide (4)

Yield: 62%. δ_H_: 9.67 (s, NH), 7.30 (d, *J* = 2.3 Hz, 1H), 7.04 (dd, *J* = 8.6, 2.3 Hz, 1H), 6.85 (d, *J* = 8.7 Hz, 1H), 4.05–3.91 (m, 4H), 3.81–3.68 (m, 4H), 3.59 (s, 8H), 2.34–2.20 (m, 1H), 1.85–1.57 (m, 5H), 1.46–1.04 (m, 5H). δ_C_: 174.2, 148.6, 144.4, 133.6, 114.6, 111.6, 106.1, 70.6, 70.5, 70.0, 69.9, 69.1, 68.9, 68.4, 45.1, 29.4, 25.6, 25.4. HRMS (ESI, positive ion mode): calc. for C_21_H_32_NO_6_ 394.2225 ([M + H]^+^), found 394.2212. HRMS (ESI, negative ion mode): calc. for C_21_H_30_NO_6_ 392.2078 ([M–H]^–^), found 392.2084.

### *N*-(2,3,5,6,8,9,11,12-octahydrobenzo[b][1,4,7,10,13]pentaoxacyclopentadecin-15-yl)-2-naphthamide (5)

Yield: 47%. δ_H_: 8.32 (s, 1H), 7.92–7.76 (m, 4H), 7.54–7.44 (m, 2H), 7.38 (d, *J* = 2.7 Hz, 1H), 7.07 (dd, *J* = 8.6, 2.5 Hz, 1H), 6.77 (d, *J* = 8.7 Hz, 1H), 4.12–3.96 (m, 4H), 3.88–3.74 (m, 4H), 3.72–3.53 (m, 8H). δ_C_: 166.8, 149.8, 146.5, 135.4, 133.2, 132.9, 132.8, 129.5, 129.1, 128.4, 128.3, 128.2, 127.4, 124.3, 115.0, 113.7, 108.0, 71.45 & 71.38, 70.95 & 70.85, 70.2, 70.0, 69.3. HRMS (ESI, positive ion mode): calc. for C_25_H_28_NO_6_ 438.1917 ([M + H]^+^), found 438.1927. HRMS (ESI, negative ion mode): calc. for C_25_H_26_NO_6_ 436.1766 ([M–H]^–^), found 436.1778.

### *N*-(2,3,5,6,8,9,11,12-octahydrobenzo[*b*][1,4,7,10,13]pentaoxacyclopentadecin-15-yl)-[1,1’-biphenyl]-4-carboxamide (6)

Yield: 38%. δ_H_: 7.79 (d, *J* = 8.3 Hz, 2H), 7.73–7.67 (m, 3H), 7.64 (s, 1H), 7.45 (t, *J* = 7.6 Hz, 3H), 7.36 (t, *J* = 7.3 Hz, 1H), 7.30 (s, 2H), 6.95 (d, *J* = 8.7 Hz, 1H), 3.79–3.73 (m, 4H), 3.61 (s, 8H). δ_C_: 165.5, 148.9, 145.5, 143.5, 139.6, 134.3, 133.5, 129.3, 128.6, 128.3, 127.2, 126.9, 114.5, 113.4, 107.8, 70.8, 70.3, 70.2, 69.4, 69.3, 69.2, 68.8. HRMS (ESI, positive ion mode): calc. for C_27_H_30_NO_6_ 464.2068 ([M + H]^+^), found 464.2083. HRMS (ESI, negative ion mode): calc. for C_27_H_28_NO_6_ 462.1922 ([M–H]^–^), found 462.1909.

### *N*-(2,3,5,6,8,9,11,12-octahydrobenzo[*b*][1,4,7,10,13]pentaoxacyclopentadecin-15-yl)pyrene-1-carboxamide (7)

Yield: 35%. δ_H_: 8.34 (d, *J* = 9.0 Hz, 1H), 8.02 (dd, *J* = 8.9, 8.2 Hz, 3H), 7.97–7.78 (m, 5H), 7.46 (s, 1H), 7.13 (d, *J* = 7.2 Hz, 1H), 6.75 (d, *J* = 8.4 Hz, 1H), 4.15–3.92 (m, 11H), 3.73 (s, 4H), 3.65–3.51 (m, 8H). δ_C_: 169.0, 147.9, 144.5, 133.2, 132.4, 128.5, 128.44, 128.38, 126.9, 126.2, 125.7, 125.6, 124.6, 124.4, 124.1, 124.01, 123.95, 114.0, 113.5, 107.1, 69.5, 69.4, 69.2, 69.1, 68.6, 68.4, 68.3, 67.7. HRMS (ESI, positive ion mode): calc. for C_31_H_30_NO_6_ 512.2068 ([M + H]^+^), found 512.2056. HRMS (ESI, negative ion mode): calc. for C_31_H_28_NO_6_ 510.1922 ([M–H]^–^), found 510.1895.

### Reference compounds with known log*P* values

Aniline (log*P* = 0.9), benzyl alcohol (log*P* = 1.1), 4-chloroaniline (log*P* = 1.8), 4-chlorophenol (log*P* = 2.4), diphenylamine (log*P* = 3.4), and diphenyl ether (log*P* = 4.2) were chosen from a list of sixty recommended reference substances. The reference substance solutions were prepared in a mixture of MeOH:H_2_O (3:1, v/v) and diluted to a sufficient concentration to allow their detection. The reference substances should normally have log*P* values which encompass the log*P* of the studied compounds.

### Solution preparation

*n*-Octanol was purchased from Sigma-Aldrich with 99% purity; double-distilled water was purchased from Corning. Salt solutions (144 mM) were prepared by dissolving the appropriate weight of each salt in the double-distilled water. The NaCl and KCl salts were dried at 120 °C for 24 h prior to weighing. Then, the obtained solutions were saturated with *n*-octanol for partitioning studies. The crown ether solutions were prepared by dissolving a known weight of each compound (**2**–**7**) in water-saturated *n*-octanol, followed by degassing by ultrasonication.

## Supplementary Material

Supplementary_Material___anonymous.docx

## Data Availability

Data associated with this study are available in the article and its supplementary material.

## Associated content

Details of the studies and copies of NMR and MS spectra for all compounds (PDF).

## Dedication

This manuscript is part of a special collection of articles dedicated to Professor Minyong Li, who passed away unexpectedly late in 2025. This special collection will serve as a perpetual memory of his friendship and many significant contributions to medicinal chemistry, chemical biology, and chemosensing.
