# Supplementary material for: A novel approach to improving solubility of drugs: chelation-assisted solubility enhancement (CHASE)
Source: J Enzyme Inhib Med Chem. 2026 Jul 27;41(1):2704738. doi: 10.1080/14756366.2026.2704738 (PMC13410548; doi:10.1080/14756366.2026.2704738)
Supplement: Supplementary_Material___anonymous.docx [file IENZ_A_2704738_SM6460.docx]

Supporting Information

**A Novel Approach to Improving Solubility of Drugs: Chelation-assisted Solubility Enhancement (CHASE)**

Table of Contents

[**Determination of partition coefficients between *n*-octanol and pure water** 2](#_Toc232445662)

[**Determination of partition coefficients between K^+^/Na^+^ solution and *n*-octanol.** 6](#_Toc232445663)

[**Copies of NMR and MS spectra** 22](#_Toc232445664)

[**References** 40](#_Toc232445665)

# **Determination of partition coefficients between *n*-octanol and pure water**

log*P* estimation between *n*-octanol and pure water was done according to OECD Guidelines for the Testing of Chemicals No. 117 (High Performance Liquid Chromatography method for log*P* estimation in range of 0 to 6, possibly extended up to 10 in exceptional cases). The guidelines were based on research by C.V. Eadsforth and P. Moser,^1^ further developed and coordinated by Umweltbundesamt of the Federal Republic of Germany.^2^

HPLC studies were conducted on Shimadzu LC2050i, using isocratic operation mode with MeOH:H_2_O (3:1, v/v, containing no trifluoroacetic acid) as the mobile phase with a flowrate of 0.5 mL/min, injection volume of 10 μL, monitoring by a UV detector at a wavelength of 210 nm (LC-2050/2060 PDA), at ambient temperature (23-25 °C). The references and investigated compounds were first solubilized in pure methanol, followed by addition of H_2_O to achieve a ratio between MeOH and H_2_O of 3 to 1 (v/v), which was similar to the HPLC mobile phase.

**Table S1** - Reference compounds with known logP values and their experimental logK values

| **Reference compounds** | **log*P^2^*** | $\boldsymbol{t}_{\boldsymbol{R}}$ **(min)** | $\boldsymbol{K=}\frac{\boldsymbol{t}_{\boldsymbol{R}}\boldsymbol{-}\boldsymbol{t}_{\boldsymbol{0}}}{\boldsymbol{t}_{\boldsymbol{0}}}$ | **log*K*** |
| --- | --- | --- | --- | --- |
| Thiourea (250 μM) | -- | 1.525 ($t_{0}$) | -- | -- |
| Aniline (20 μM) | 0.9 | 1.867 | 0.2243 | -0.65 |
| Benzyl alcohol (200 μM) | 1.1 | 1.941 | 0.2728 | -0.56 |
| 4-Chloroaniline (100 μM) | 1.8 | 2.171 | 0.4236 | -0.37 |
| 4-Chlorophenol (20 μM) | 2.4 | 2.443 | 0.6020 | -0.22 |
| Diphenylamine (200 μM) | 3.4 | 4.266 | 1.7974 | 0.25 |
| Diphenyl ether (10 μM) | 4.2 | 6.869 | 3.5043 | 0.54 |

Seven reference compounds with known log*P* values from a reference list were used to measure their retention times $t_{R}$ on HPLC column. Since thiourea is unretained, its retention time is defined as dead time $t_{0}$.^2^ The retention times were then expressed through the capacity factor ($K=\frac{t_{R}-t_{0}}{t_{0}}$). All the retention times, capacity factors, reference log*P* values and calculated log*K* values were presented in **Table S1**. With log*K* and known log*P* values, a calibration curve was built to first determine linear regression coefficients *a* and *b*, based on **Equation I**.

$logP=a+b*logK$ (I)


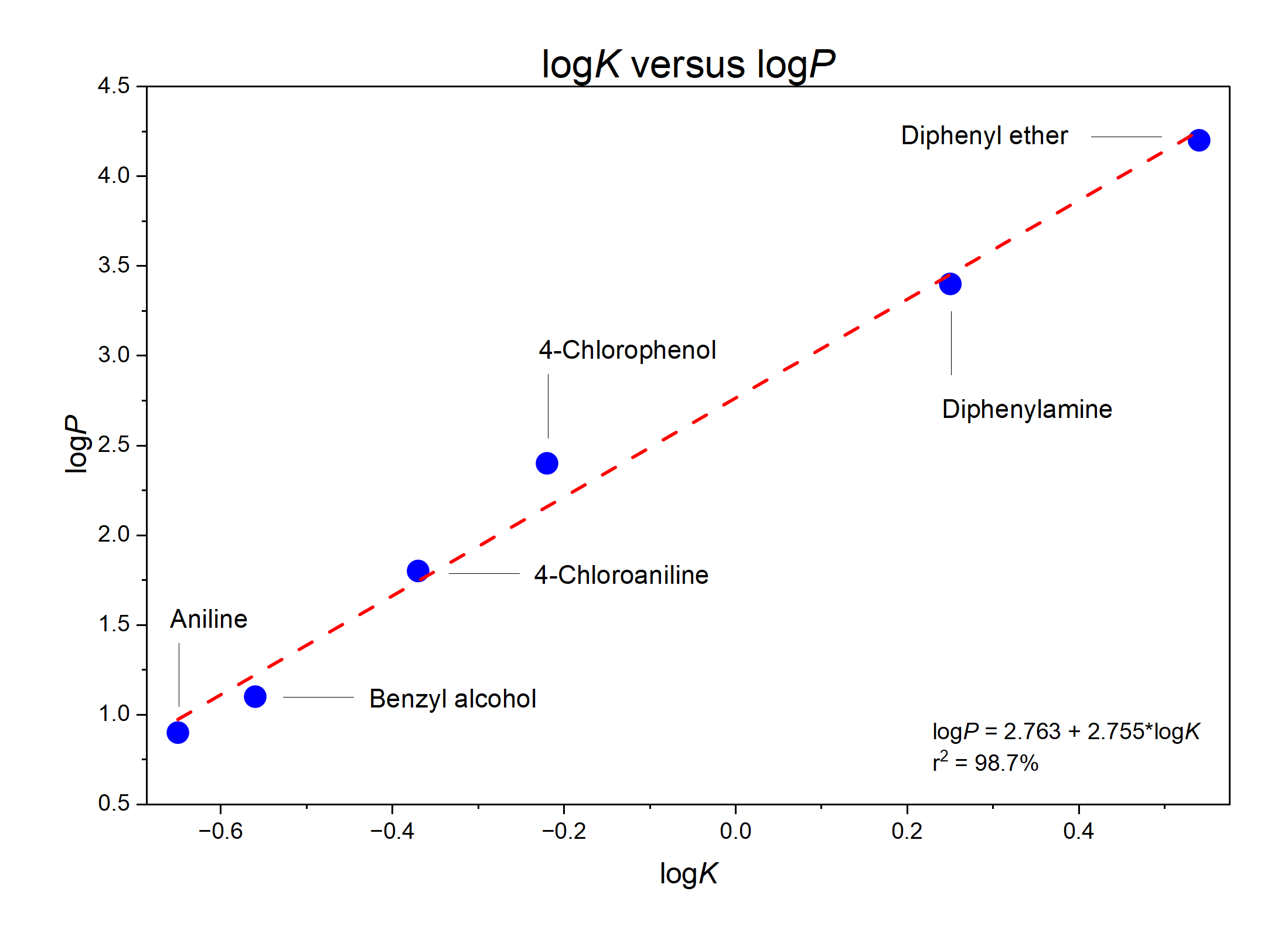


**Figure S1** – A standard curve establishing a linear relationship between logK and logP values

**Table S2** – Investigated crown ethers and their experimental logK values

| **Investigated compounds** | **log*P determined from Std. Curve*** | $\boldsymbol{t}_{\boldsymbol{R}}$ **(min)** | $\boldsymbol{K=}\frac{\boldsymbol{t}_{\boldsymbol{R}}\boldsymbol{-}\boldsymbol{t}_{\boldsymbol{0}}}{\boldsymbol{t}_{\boldsymbol{0}}}$ | **log*K*** |
| --- | --- | --- | --- | --- |
| Thiourea (250 μM) | -- | 1.525 (*t*_0_) | -- | -- |
| Compound **2** (200 μM) | 1.62 | 2.112 | 0.3849 | -0.415 |
|  | 1.63 | 2.114 | 0.3862 | -0.413 |
|  | 1.62 | 2.112 | 0.3849 | -0.415 |
|  | On average, log*P* = 1.62 ± 0.01 | | | |
| Compound **3** (125 μM) | 2.29 | 2.550 | 0.6721 | -0.173 |
|  | 2.29 | 2.553 | 0.6741 | -0.171 |
|  | 2.31 | 2.565 | 0.6820 | -0.166 |
|  | On average, log*P* = 2.30 ± 0.01 | | | |
| Compound **4** (125 μM) | 2.29 | 2.558 | 0.6773 | -0.169 |
|  | 2.29 | 2.549 | 0.6715 | -0.173 |
|  | 2.31 | 2.570 | 0.6852 | -0.164 |
|  | On average, log*P* = 2.30 ± 0.01 | | | |
| Compound **5** (125 μM) | 2.72 | 3.002 | 0.9685 | -0.014 |
|  | 2.73 | 3.013 | 0.9757 | -0.019 |
|  | 2.72 | 2.992 | 0.9620 | -0.017 |
|  | On average, log*P* = 2.72 ± 0.01 | | | |
| Compound **6** (200 μM) | 3.25 | 3.814 | 1.501 | 0.176 |
|  | 3.26 | 3.829 | 1.511 | 0.179 |
|  | 3.26 | 3.826 | 1.509 | 0.179 |
|  | On average, log*P* = 3.25 ± 0.01 | | | |
| Compound **7** (125 μM) | 3.61 | 4.609 | 2.022 | 0.305 |
|  | 3.62 | 4.635 | 2.039 | 0.309 |
|  | 3.61 | 4.608 | 2.022 | 0.305 |
|  | On average, log*P* = 3.61 ± 0.01 | | | |

$logP=2.763+2.755*logK$ (II)

The octanol-water partition coefficient of a test compound can be computed from its own retention time, capacity factor *K* and then log*K* value. Imputing log*K* value into **Equation II** allows determination of corresponding experimental partition coefficient (log*P*). Experiments were performed three times. Measured data of the investigated compounds is summarized in **Table S2**.

**Figure S2** – Seven reference compounds for building the standard curve

**Figure S3** – Six model compounds for studies of partitioning

# **Determination of partition coefficients between K^+^/Na^+^ solution and *n*-octanol.**

log*P* estimation between *n*-octanol and solution of K^+^/Na^+^ was done according to OECD Guidelines for the Testing of Chemicals No. 107 (shake flask method).^3^ The guidelines were based on principle of Nernst distribution law. Compounds investigated were allowed to partition between two phases, with one phase being *n*-octanol and the other phase being either K^+^ 144 mM solution or Na^+^ 144 mM solution. Then, concentration of each phase was measured based on standard curves to determine a partition coefficient. For analysis, the HPLC operation mode was gradient, using acetonitrile:water (5% to 95%, v/v) typically as the mobile phase with a flowrate of 0.5 mL/min, monitored by a UV detector at a wavelength of 210 nm.

Before partitioning, two phases were mutually saturated by shaking stock bottles at ambient temperature for at least 24 hours on a mechanical shaker. Four stock bottles were prepared: 1) one bottle containing *n*-octanol and a sufficient amount of K^+^ 144 mM solution, 2) one containing *n*-octanol and a sufficient amount of Na^+^ 144 mM solution, 3) one containing K^+^ 144 mM solution and a sufficient amount of *n*-octanol, and 4) the other containing Na^+^ 144 mM solution and a sufficient amount of *n*-octanol. It was followed by phase separation by centrifugation at room temperature (25 °C), 5000 rpm for 5 minutes.

The partition coefficient, *P*, is defined as the ratio of the equilibrium concentrations of a dissolved substance in a two-phase system:

$P=\frac{{[analyte]}_{oct.}}{{[analyte]}_{aq.}}$ (III)

***N*-(2,3,5,6,8,9,11,12-octahydrobenzo[*b*][1,4,7,10,13]pentaoxacyclopentadecin-15-yl)-benzamide (2)**

**-Standard curve in pure methanol:**


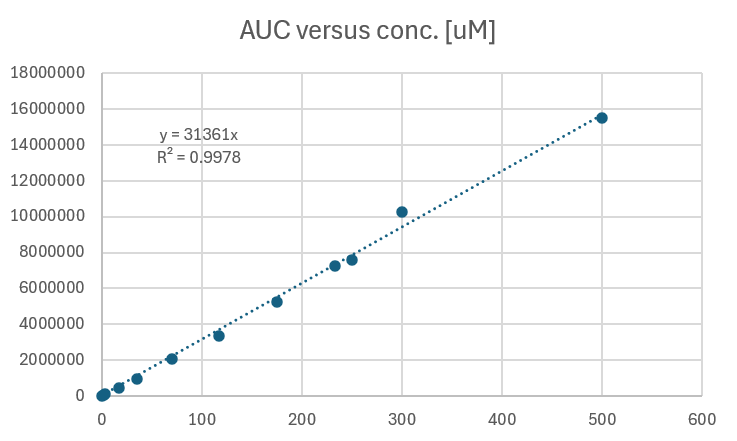


**Figure S4** - Standard curve of compound **2** in methanol

A standard curve was built based on HPLC area under the curve (AUC) to measure the amount of the analyte **2**. Samples were prepared from 0 uM [blank] to 500 μM, in pure methanol.

**-Partitioning of compound 2 between *n*-octanol and K^+^ 144 mM solution:**

A solution of crown ether was prepared in *n*-octanol pre-saturated with K^+^ 144mM solution. Then 3 mL octanol solution (325 μM of the analyte) was added to a centrifuge tube with 3 mL K^+^ 144mM solution (no analyte). The tube was shaken overnight, followed by centrifugation at room temperature (25 °C), 5000 rpm for 5 minutes. Phases were separated and stored in different glass vials for HPLC analysis. Experiment was triplicated.

**Table S3** – Experimental results of partitioning between n-octanol and K^+^ aqueous phases

| Run 1 | Octanol phase | | Aqueous phase |
| --- | --- | --- | --- |
| AUC | 9’796’896 | | 428’339 |
| Conc. [uM] | 312.4 | | 13.7 |
| *P* | 22.8 | | |
| log*P* | 1.36 | | |
| **Run 2** | **Octanol phase** | **Aqueous phase** | |
| AUC | 9’739’047 | 432’565 | |
| Conc. [uM] | 310.5 | 13.8 | |
| *P* | 22.5 | | |
| log*P* | 1.35 | | |
| **Run 3** | **Octanol phase** | **Aqueous phase** | |
| AUC | 9’540’403 | 429’830 | |
| Conc. [uM] | 304.2 | 13.7 | |
| *P* | 22.2 | | |
| log*P* | 1.35 | | |
| On average | *P* = 22.6 ± 0.2  log*P* = 1.35 ± 0.01 | | |

**-Partitioning of compound 2 between *n*-octanol and Na^+^ 144 mM solution:**

A solution of crown ether was prepared in *n*-octanol pre-saturated with Na^+^ 144mM solution. Then 3 mL octanol solution (305 μM of the analyte) was added to a centrifuge tube with 3 mL Na^+^ 144mM solution (no analyte). The tube was shaken overnight, followed by centrifugation at room temperature, 5000 rpm for 5 minutes. Phases were separated and stored in different glass vials for HPLC analysis. Experiment was triplicated.

**Table S4** - Experimental results of partitioning between n-octanol and Na^+^ aqueous phases

| Run 1 | Octanol phase | | Aqueous phase |
| --- | --- | --- | --- |
| AUC | 9’441’833 | | 400’669 |
| Conc. [uM] | 301.1 | | 12.8 |
| *P* | 23.5 | | |
| log*P* | 1.37 | | |
| **Run 2** | **Octanol phase** | **Aqueous phase** | |
| AUC | 9’360’568 | 384’182 | |
| Conc. [uM] | 298.5 | 12.3 | |
| *P* | 24.3 | | |
| log*P* | 1.39 | | |
| **Run 3** | **Octanol phase** | **Aqueous phase** | |
| AUC | 9’347’447 | 403’912 | |
| Conc. [uM] | 298.1 | 12.9 | |
| *P* | 23.1 | | |
| log*P* | 1.36 | | |
| On average | *P* = 23.6 ± 0.5  log*P* = 1.37 ± 0.01 | | |

**-Partitioning of compound 2 between *n*-octanol and pure water:**

A solution of crown ether was prepared in *n*-octanol pre-saturated with Na^+^ 144mM solution. Then 3 mL octanol solution (225 μM of the analyte) was added to a centrifuge tube with 3 mL pure water. The tube was shaken overnight, followed by centrifugation at room temperature, 5000 rpm for 5 minutes. Phases were separated and stored in different glass vials for HPLC analysis. Experiment was triplicated.

**Table S5** - Experimental results of partitioning between n-octanol and Na^+^ aqueous phases

| Run 1 | Octanol phase | | Aqueous phase |
| --- | --- | --- | --- |
| AUC | 6’832’398 | | 184’613 |
| Conc. [uM] | 217.9 | | 5.9 |
| *P* | 36.9 | | |
| log*P* | 1.57 | | |
| **Run 2** | **Octanol phase** | **Aqueous phase** | |
| AUC | 6’767’406 | 179’984 | |
| Conc. [uM] | 215.8 | 5.7 | |
| *P* | 37.9 | | |
| log*P* | 1.59 | | |
| **Run 3** | **Octanol phase** | **Aqueous phase** | |
| AUC | 6’854’305 | 184’288 | |
| Conc. [uM] | 218.6 | 5.9 | |
| *P* | 37.2 | | |
| log*P* | 1.57 | | |
| On average | *P* = 37.3 ± 0.5  log*P* = 1.58 ± 0.01 | | |

***N*-(2,3,5,6,8,9,11,12-octahydrobenzo[*b*][1,4,7,10,13]pentaoxacyclopentadecin-15-yl)hexamide** (**3**)

**-Standard curve in pure methanol:**


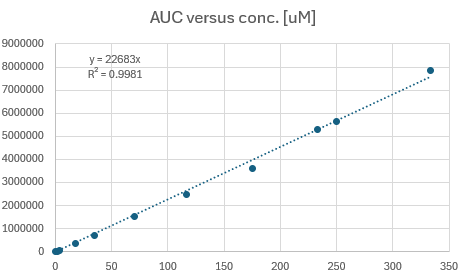


**Figure S5** - Standard curve of compound **3** in methanol

A standard curve was built based on HPLC area under the curve (AUC) to measure the amount of the analyte **3**. Samples were prepared from 0 uM [blank] to 333.3 μM, in pure methanol.

**-Partitioning of compound 3 between *n*-octanol and K^+^ 144 mM solution:**

A solution of crown ether was prepared in *n*-octanol pre-saturated with K^+^ 144mM solution. Then 2.5 mL octanol solution (270 μM of the analyte) was added to a centrifuge tube with 2.5 mL K^+^ 144mM solution (no analyte). The tube was shaken overnight, followed by centrifugation at room temperature, 5000 rpm for 5 minutes. Phases were separated and stored in different vials for HPLC analysis. Experiment was triplicated.

**Table S6** – Experimental results of partitioning between n-octanol and K^+^ aqueous phases

| Run 1 | Octanol phase | | Aqueous phase |
| --- | --- | --- | --- |
| AUC | 5’933’834 | | 121’247 |
| Conc. [uM] | 261.6 | | 5.3 |
| *P* | 49.3 | | |
| log*P* | 1.69 | | |
| **Run 2** | **Octanol phase** | **Aqueous phase** | |
| AUC | 5’948’535 | 119’871 | |
| Conc. [uM] | 262.2 | 5.3 | |
| *P* | 49.5 | | |
| log*P* | 1.69 | | |
| **Run 3** | **Octanol phase** | **Aqueous phase** | |
| AUC | 5’948’249 | 113’677 | |
| Conc. [uM] | 262.2 | 5.0 | |
| *P* | 52.4 | | |
| log*P* | 1.72 | | |
| On average | *P* = 50.4 ± 1.4  log*P* = 1.70 ± 0.01 | | |

**-Partitioning of compound 3 between *n*-octanol and Na^+^ 144 mM solution:**

A solution of crown ether was prepared in *n*-octanol pre-saturated with Na^+^ 144mM solution. Then 2.5 mL octanol solution (270 μM of the analyte) was added to a centrifuge tube with 2.5 mL Na^+^ 144mM solution (no analyte). The tube was shaken overnight, followed by centrifugation at room temperature, 5000 rpm for 5 minutes. Phases were separated and stored in different vials for HPLC analysis. Experiment was triplicated.

**Table S7** - Experimental results of partitioning between n-octanol and Na^+^ aqueous phases

| Run 1 | Octanol phase | | Aqueous phase |
| --- | --- | --- | --- |
| AUC | 5’994’156 | | 105’276 |
| Conc. [uM] | 264.3 | | 4.6 |
| *P* | 57.5 | | |
| log*P* | 1.76 | | |
| **Run 2** | **Octanol phase** | **Aqueous phase** | |
| AUC | 5’993’834 | 111’751 | |
| Conc. [uM] | 264.2 | 4.9 | |
| *P* | 53.9 | | |
| log*P* | 1.73 | | |
| **Run 3** | **Octanol phase** | **Aqueous phase** | |
| AUC | 6’009’459 | 104’496 | |
| Conc. [uM] | 264.9 | 4.6 | |
| *P* | 57.6 | | |
| log*P* | 1.76 | | |
| On average | *P* = 56.3 ± 1.7  log*P* = 1.75 ± 0.01 | | |

***N*-(2,3,5,6,8,9,11,12-octahydrobenzo[*b*][1,4,7,10,13]pentaoxacyclopentadecin-15-yl)cyclohexanecarboxamide** (**4**)

**-Standard curve in pure methanol:**


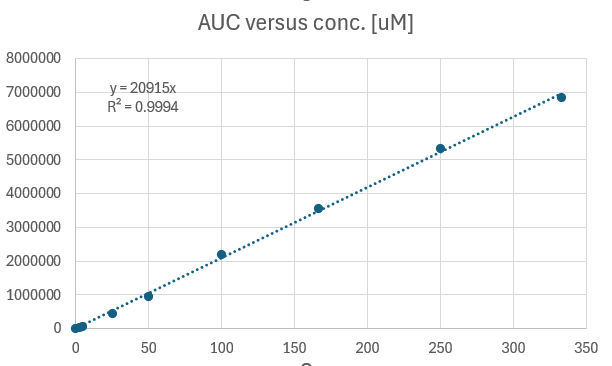


**Figure S6** - Standard curve of compound **4** in methanol

A standard curve was built based on HPLC area under the curve (AUC) to measure the amount of the analyte **4**. Samples were prepared from 0 uM [blank] to 333.3 μM, in pure methanol.

**-Partitioning of compound 4 between *n*-octanol and K^+^ 144 mM solution:**

A solution of crown ether was prepared in *n*-octanol pre-saturated with K^+^ 144mM solution. Then 5 mL octanol solution (295 μM of the analyte) was added to a centrifuge tube with 5 mL K^+^ 144mM solution (no analyte). The tube was shaken overnight, followed by centrifugation at room temperature, 5000 rpm for 5 minutes. Phases were separated and stored in different glass vials for HPLC analysis. Experiment was triplicated.

**Table S8** – Experimental results of partitioning between n-octanol and K^+^ aqueous phases

| Run 1 | Octanol phase | | Aqueous phase |
| --- | --- | --- | --- |
| AUC | 5’955’724 | | 126’907 |
| Conc. [uM] | 284.8 | | 6.1 |
| *P* | 46.7 | | |
| log*P* | 1.67 | | |
| **Run 2** | **Octanol phase** | **Aqueous phase** | |
| AUC | 5’961’535 | 126’122 | |
| Conc. [uM] | 285.0 | 6.0 | |
| *P* | 47.5 | | |
| log*P* | 1.68 | | |
| **Run 3** | **Octanol phase** | **Aqueous phase** | |
| AUC | 5’959’009 | 125’023 | |
| Conc. [uM] | 284.9 | 6.0 | |
| *P* | 47.5 | | |
| log*P* | 1.68 | | |
| On average | *P* = 47.2 ± 0.4  log*P* = 1.67 ± 0.01 | | |

**-Partitioning of compound 4 between *n*-octanol and Na^+^ 144 mM solution:**

A solution of crown ether was prepared in *n*-octanol pre-saturated with Na^+^ 144mM solution. Then 5 mL octanol solution (290 μM of the analyte) was added to a centrifuge tube with 5 mL Na^+^ 144mM solution (no analyte). The tube was shaken overnight, followed by centrifugation at room temperature, 5000 rpm for 5 minutes. Phases were separated and stored in different glass vials for HPLC analysis. Experiment was triplicated.

**Table S9** – Experimental results of partitioning between n-octanol and Na^+^ aqueous phases

| Run 1 | Octanol phase | | Aqueous phase |
| --- | --- | --- | --- |
| AUC | 5’881’279 | | 119’572 |
| Conc. [uM] | 281.2 | | 5.7 |
| *P* | 49.3 | | |
| log*P* | 1.69 | | |
| **Run 2** | **Octanol phase** | **Aqueous phase** | |
| AUC | 5’892’288 | 117’354 | |
| Conc. [uM] | 281.8 | 5.6 | |
| *P* | 50.3 | | |
| log*P* | 1.70 | | |
| **Run 3** | **Octanol phase** | **Aqueous phase** | |
| AUC | 5’884’036 | 118’894 | |
| Conc. [uM] | 281.3 | 5.7 | |
| *P* | 49.4 | | |
| log*P* | 1.69 | | |
| On average | *P* = 49.7 ± 0.4  log*P* = 1.70 ± 0.01 | | |

***N*-(2,3,5,6,8,9,11,12-octahydrobenzo[b][1,4,7,10,13]pentaoxacyclopentadecin-15-yl)-2-naphthamide** (**5**)

**-Standard curve in pure methanol:**


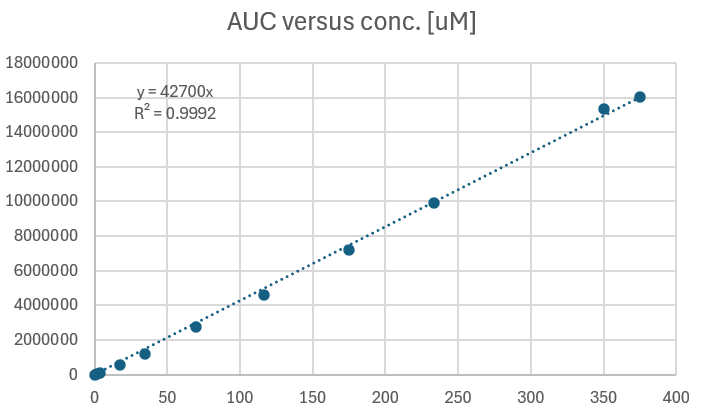


**Figure S7** - Standard curve of compound **5** in methanol

A standard curve was built based on HPLC area under the curve (AUC) to measure the amount of the analyte **5**. Samples were prepared from 0 uM [blank] to 375.0 μM, in pure methanol.

**-Partitioning of compound 5 between *n*-octanol and K^+^ 144 mM solution:**

A solution of crown ether was prepared in *n*-octanol pre-saturated with K^+^ 144mM solution. Then 3 mL octanol solution of analyte was added to a centrifuge tube with 3 mL K^+^ 144mM solution (no analyte). The tube was shaken overnight, followed by centrifugation at room temperature, 5000 rpm for 5 minutes. Phases were separated and stored in different glass vials for HPLC analysis. Experiment was triplicated.

**Table S10** – Experimental results of partitioning between n-octanol and K^+^ aqueous phases

| Run 1 | Octanol phase | | Aqueous phase |
| --- | --- | --- | --- |
| AUC | 12’225’218 | | 461’762 |
| Conc. [uM] | 286.3 | | 10.8 |
| *P* | 26.5 | | |
| log*P* | 1.42 | | |
| **Run 2** | **Octanol phase** | **Aqueous phase** | |
| AUC | 12’669’452 | 460’130 | |
| Conc. [uM] | 296.7 | 10.8 | |
| *P* | 27.5 | | |
| log*P* | 1.44 | | |
| **Run 3** | **Octanol phase** | **Aqueous phase** | |
| AUC | 12’365’559 | 460’992 | |
| Conc. [uM] | 289.6 | 10.8 | |
| *P* | 26.8 | | |
| log*P* | 1.43 | | |
| On average | *P* = 26.9 ± 0.4  log*P* = 1.43 ± 0.01 | | |

**-Partitioning of compound 5 between *n*-octanol and Na^+^ 144 mM solution:**

A solution of crown ether was prepared in *n*-octanol pre-saturated with Na^+^ 144mM solution. Then 5 mL octanol solution (375 μM of the analyte) was added to a centrifuge tube with 5 mL Na^+^ 144mM solution (no analyte). The tube was shaken overnight, followed by centrifugation at room temperature, 5000 rpm for 5 minutes. Phases were separated and stored in different glass vials for HPLC analysis. Experiment was triplicated.

**Table S11** – Experimental results of partitioning between n-octanol and Na^+^ aq. phases

| Run 1 | Octanol phase | | Aqueous phase |
| --- | --- | --- | --- |
| AUC | 15’720’711 | | 208’301 |
| Conc. [uM] | 368.2 | | 4.9 |
| *P* | 75.1 | | |
| log*P* | 1.88 | | |
| **Run 2** | **Octanol phase** | **Aqueous phase** | |
| AUC | 15’405’084 | 195’099 | |
| Conc. [uM] | 360.8 | 4.6 | |
| *P* | 78.4 | | |
| log*P* | 1.89 | | |
| **Run 3** | **Octanol phase** | **Aqueous phase** | |
| AUC | 15’818’887 | 228’981 | |
| Conc. [uM] | 370.5 | 5.4 | |
| *P* | 68.6 | | |
| log*P* | 1.84 | | |
| On average | *P* = 74.0 ± 4.1  log*P* = 1.87 ± 0.02 | | |

***N*-(2,3,5,6,8,9,11,12-octahydrobenzo[*b*][1,4,7,10,13]pentaoxacyclopentadecin-15-yl)-[1,1'-biphenyl]-4-carboxamide** (**6**).

**-Standard curve in pure methanol:**


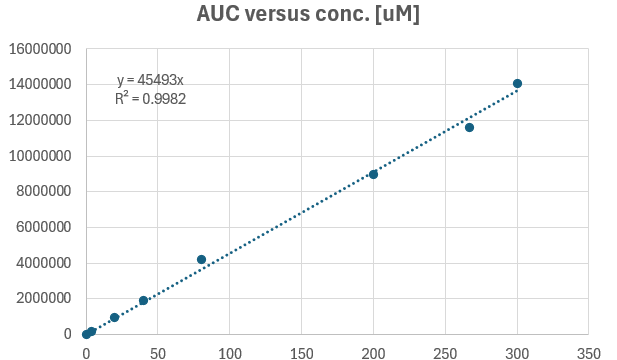


**Figure S8** - Standard curve of compound **6** in methanol

A standard curve was built based on HPLC area under the curve (AUC) to measure the amount of the analyte **6**. Samples were prepared from 0 uM [blank] to 300.0 μM, in pure methanol.

**-Partitioning of compound 6 between *n*-octanol and K^+^ 144 mM solution:**

A solution of crown ether was prepared in *n*-octanol pre-saturated with K^+^ 144mM solution. Then 3 mL octanol solution (260 μM of the analyte) was added to a centrifuge tube with 3 mL K^+^ 144mM solution (no analyte). The tube was shaken overnight, followed by centrifugation at room temperature, 5000 rpm for 5 minutes. Phases were separated and stored in different glass vials for HPLC analysis. Experiment was triplicated.

**Table S12** – Experimental results of partitioning between n-octanol and K^+^ aq. phases

| Run 1 | Octanol phase | | Aqueous phase |
| --- | --- | --- | --- |
| AUC | 11’809’573 | | 15’887 |
| Conc. [uM] | 259.6 | | 0.35 |
| *P* | 742 | | |
| log*P* | 2.87 | | |
| **Run 2** | **Octanol phase** | **Aqueous phase** | |
| AUC | 11’476’903 | 17’830 | |
| Conc. [uM] | 252.3 | 0.39 | |
| *P* | 647 | | |
| log*P* | 2.81 | | |
| **Run 3** | **Octanol phase** | **Aqueous phase** | |
| AUC | 11’614’591 | 15’530 | |
| Conc. [uM] | 255.3 | 0.34 | |
| *P* | 751 | | |
| log*P* | 2.88 | | |
| On average | *P* = 713 ± 58  log*P* = 2.85 ± 0.04 | | |

**-Partitioning of compound 6 between *n*-octanol and Na^+^ 144 mM solution:**

A solution of crown ether was prepared in *n*-octanol pre-saturated with Na^+^ 144mM solution. Then 3 mL octanol solution (261 μM of the analyte) was added to a centrifuge tube with 3 mL Na^+^ 144mM solution (no analyte). The tube was shaken overnight, followed by centrifugation at room temperature, 5000 rpm for 5 minutes. Phases were separated and stored in different glass vials for HPLC analysis. Experiment was triplicated.

**Table S13** – Experimental results of partitioning between n-octanol and Na^+^ aq. phases

| Run 1 | Octanol phase | | Aqueous phase |
| --- | --- | --- | --- |
| AUC | 11’853’095 | | 14’900 |
| Conc. [uM] | 260.5 | | 0.33 |
| *P* | 796 | | |
| log*P* | 2.90 | | |
| **Run 2** | **Octanol phase** | **Aqueous phase** | |
| AUC | 11’927’206 | 15’681 | |
| Conc. [uM] | 262.2 | 0.34 | |
| *P* | 761 | | |
| log*P* | 2.88 | | |
| **Run 3** | **Octanol phase** | **Aqueous phase** | |
| AUC | 11’786’713 | 13’476 | |
| Conc. [uM] | 259.1 | 0.3 | |
| *P* | 875 | | |
| log*P* | 2.94 | | |
| On average | *P* = 811 ± 58  log*P* = 2.91 ± 0.03 | | |

***N*-(2,3,5,6,8,9,11,12-octahydrobenzo[*b*][1,4,7,10,13]pentaoxacyclopentadecin-15-yl)pyrene-1-carboxamide** (**7**)

**-Partitioning of compound 7 between *n*-octanol and K^+^ 144 mM solution:**


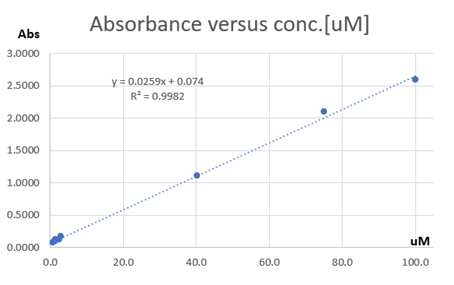


**Figure S9** - Standard curve of compound **7** in octanol

A standard curve was built using UV-Vis spectrometry to measure the UV absorbance of the analyte **7** at λ_max_. Samples were prepared in K^+^ solution-saturated octanol, with concentration in range of 0.7 – 100 μM.


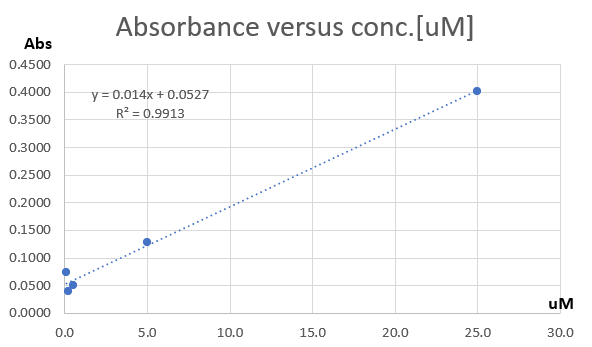


**Figure S10** - Standard curve of compound **7** in K^+^ 144 mM solution

A standard curve was built using UV-Vis spectrometry to measure the UV absorbance of the analyte **7** at λ_max_. Samples were prepared in octanol-saturated aqueous K^+^ 144 mM solution, with concentration in range of 0.12 – 25.0 μM.

A solution of crown ether was prepared in *n*-octanol pre-saturated with K^+^ 144mM solution. Then 5 mL octanol solution (88 μM of the analyte) was added to a centrifuge tube with 5 mL K^+^ 144mM solution (no analyte). The tube was shaken overnight, followed by centrifugation at room temperature, 5000 rpm for 5 minutes. Phases were separated and stored in different glass vials for UV-Vis analysis. Experiment was triplicated.

**Table S14** – Experimental results of partitioning between n-octanol and K^+^ aq. phases

| Run 1 | Octanol phase | | Aqueous phase |
| --- | --- | --- | --- |
| Absorbance | 2.0885 | | 0.1650 |
| Conc. [uM] | 77.8 | | 8.0 |
| *P* | 9.7 | | |
| log*P* | 0.99 | | |
| **Run 2** | **Octanol phase** | **Aqueous phase** | |
| Absorbance | 2.0701 | 0.1622 | |
| Conc. [uM] | 77.1 | 7.8 | |
| *P* | 9.9 | | |
| log*P* | 1.00 | | |
| **Run 3** | **Octanol phase** | **Aqueous phase** | |
| Absorbance | 2.0975 | 0.1678 | |
| Conc. [uM] | 78.1 | 8.2 | |
| *P* | 9.5 | | |
| log*P* | 0.98 | | |
| On average | *P* = 9.7 ± 0.2  log*P* = 0.99 ± 0.01 | | |

**-Partitioning of compound 7 between *n*-octanol and Na^+^ 144 mM solution:**


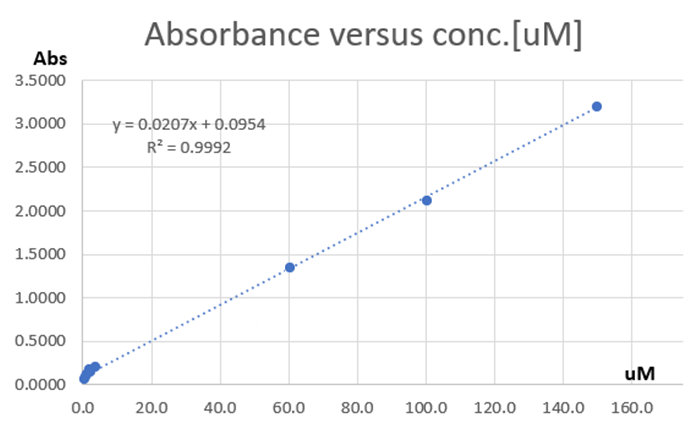


**Figure S11** - Standard curve of compound **7** in octanol

A standard curve was built using UV-Vis spectrometry to measure the UV absorbance of the analyte **7** at λ_max_. Samples were prepared in Na^+^ solution-saturated octanol, with concentration in range of 0.60 – 150 μM.


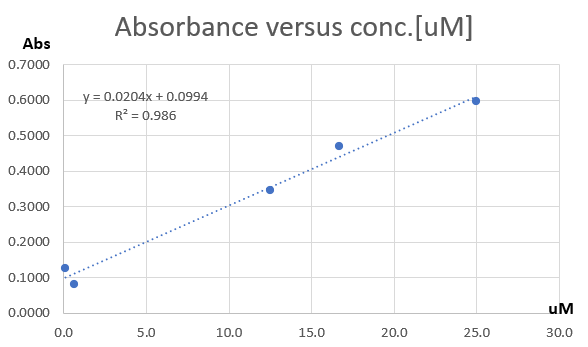


**Figure S12** - Standard curve of compound **7** in Na^+^ 144 mM solution

A standard curve was built using UV-Vis spectrometry to measure the UV absorbance of the analyte **7** at λ_max_. Samples were prepared in octanol-saturated aqueous Na^+^ 144 mM solution, with concentration in range of 0.10 – 25.0 μM.

A solution of crown ether was prepared in *n*-octanol pre-saturated with Na^+^ 144mM solution. Then 5 mL octanol solution (100 μM of the analyte) was added to a centrifuge tube with 5 mL Na^+^ 144mM solution (no analyte). The tube was shaken overnight, followed by centrifugation at room temperature, 5000 rpm for 5 minutes. Phases were separated and stored in different glass vials for analysis. Experiment was triplicated.

**Table S15** – Experimental results of partitioning between n-octanol and Na^+^ aq. phases

| Run 1 | Octanol phase | | Aqueous phase |
| --- | --- | --- | --- |
| Absorbance | 2.0610 | | 0.1682 |
| Conc. [uM] | 95.0 | | 3.4 |
| *P* | 27.9 | | |
| log*P* | 1.45 | | |
| **Run 2** | **Octanol phase** | **Aqueous phase** | |
| Absorbance | 2.0692 | 0.1699 | |
| Conc. [uM] | 95.4 | 3.5 | |
| *P* | 27.3 | | |
| log*P* | 1.44 | | |
| **Run 3** | **Octanol phase** | **Aqueous phase** | |
| Absorbance | 2.0734 | 0.1696 | |
| Conc. [uM] | 95.6 | 3.4 | |
| *P* | 28.1 | | |
| log*P* | 1.45 | | |
| On average | *P* = 27.8 ± 0.4  log*P* = 1.45 ± 0.01 | | |

# **Copies of NMR and MS spectra**


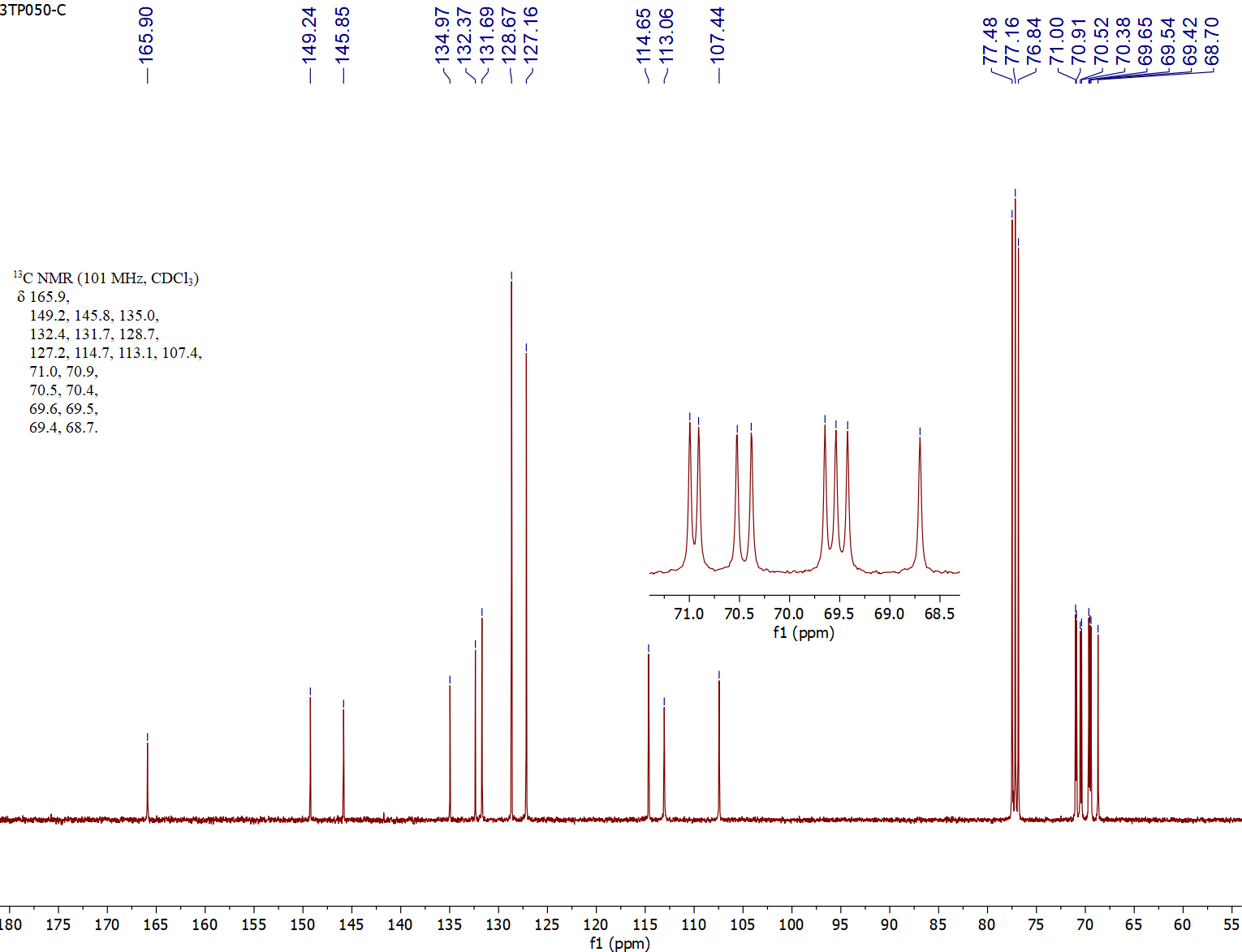


Compound **2**

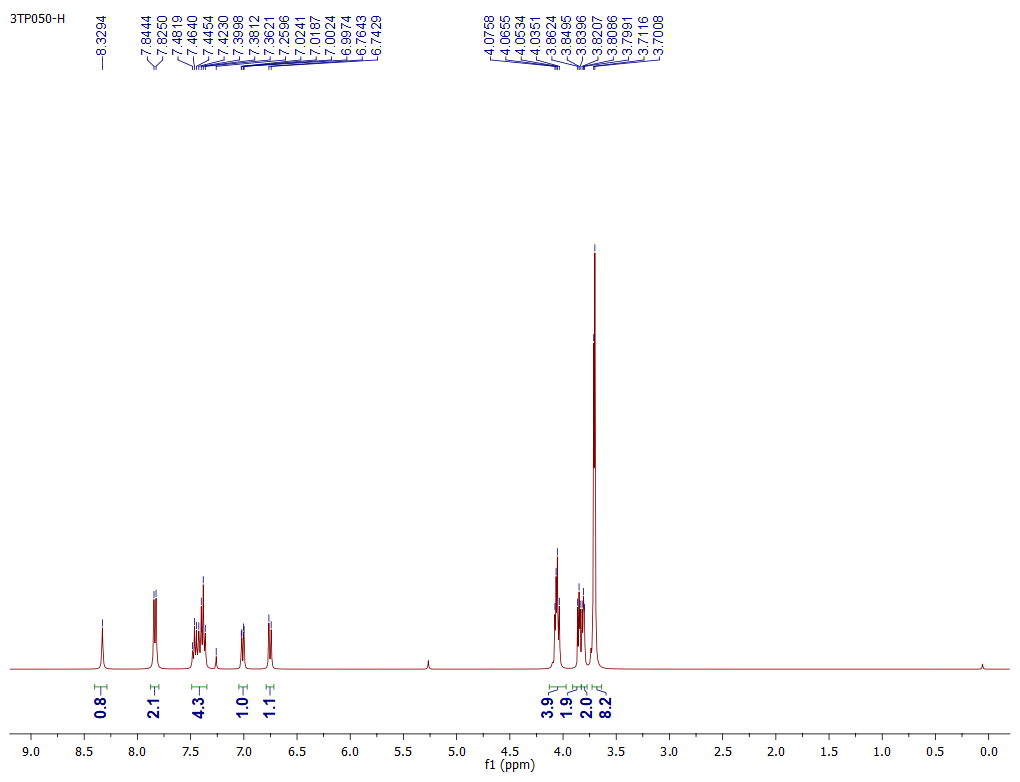


Compound **2**

Exact MW: **387.1682** g/mol

Exact MW: **387.1682** g/mol


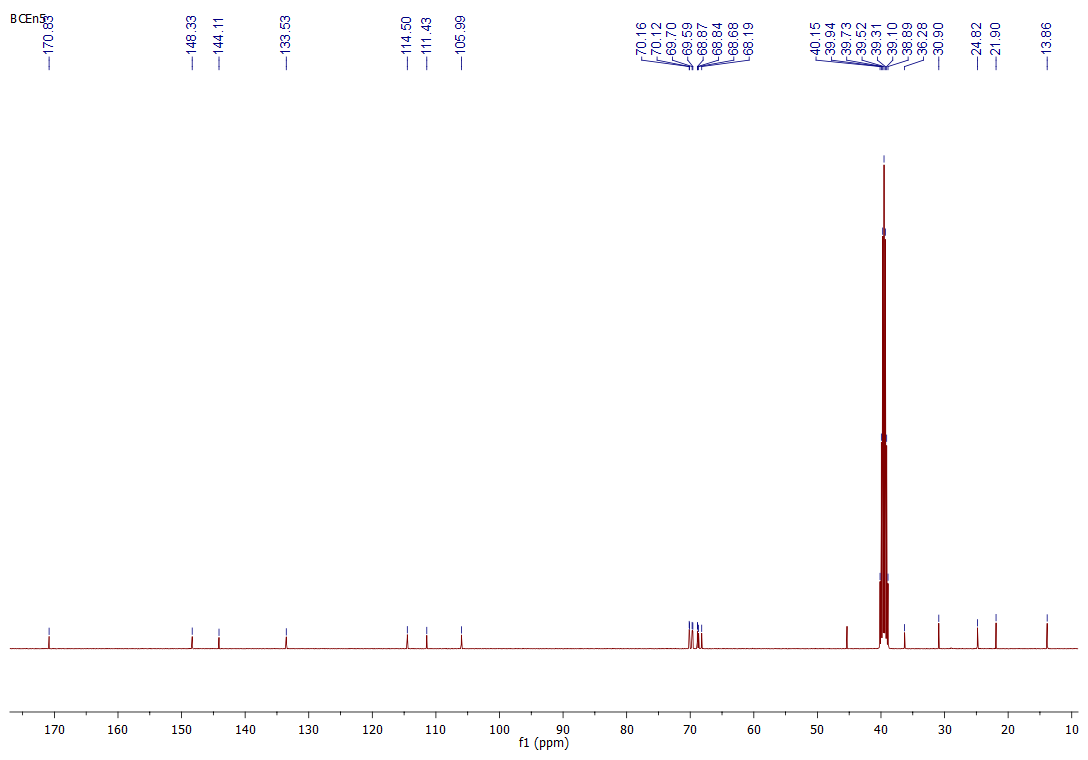


Compound **3**

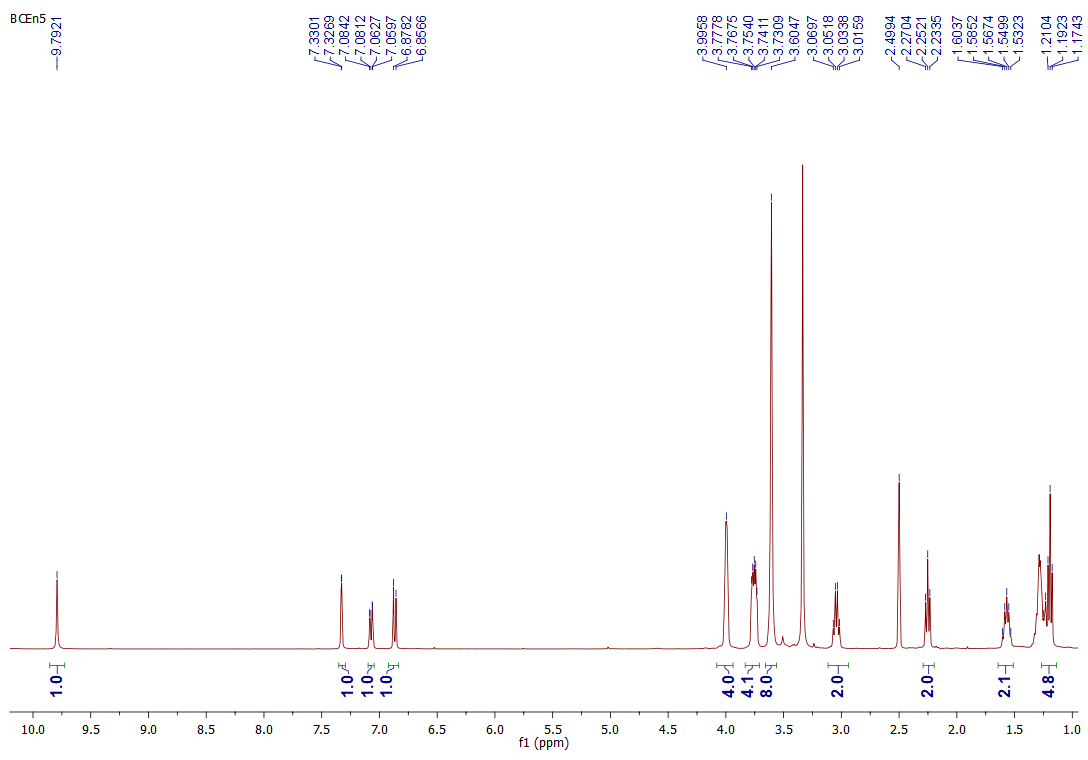


Compound **3**

Exact MW: **381.2151** g/mol

Exact MW: **381.2151** g/mol

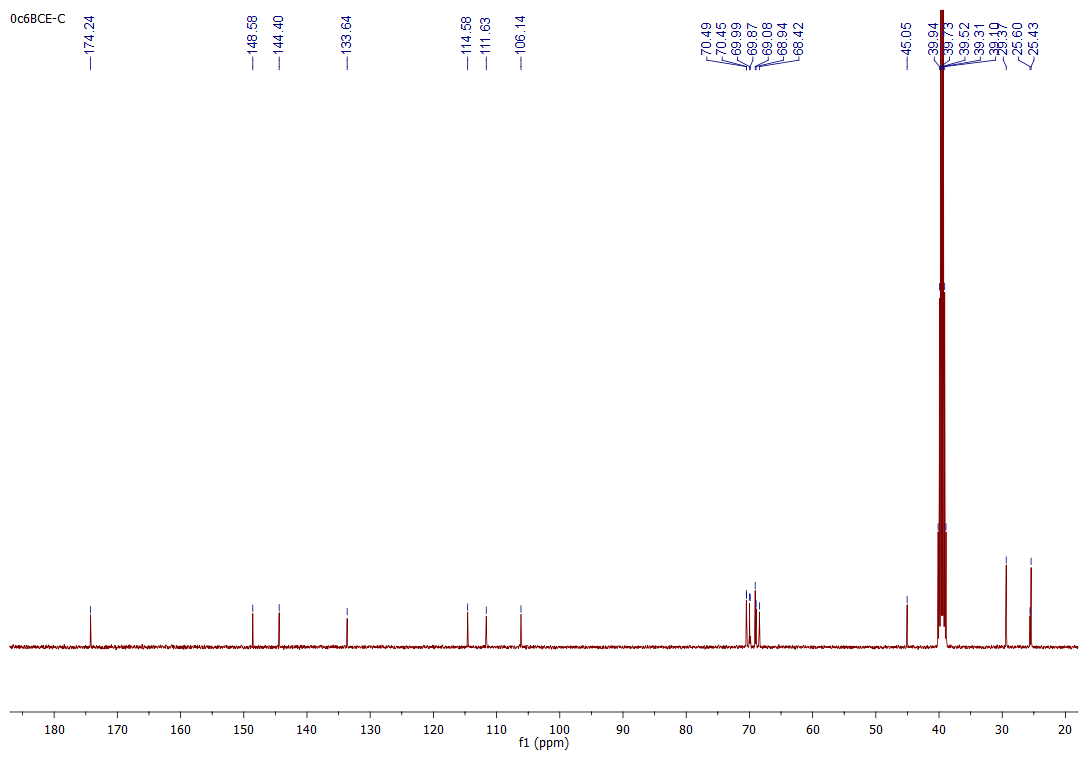


Compound **4**

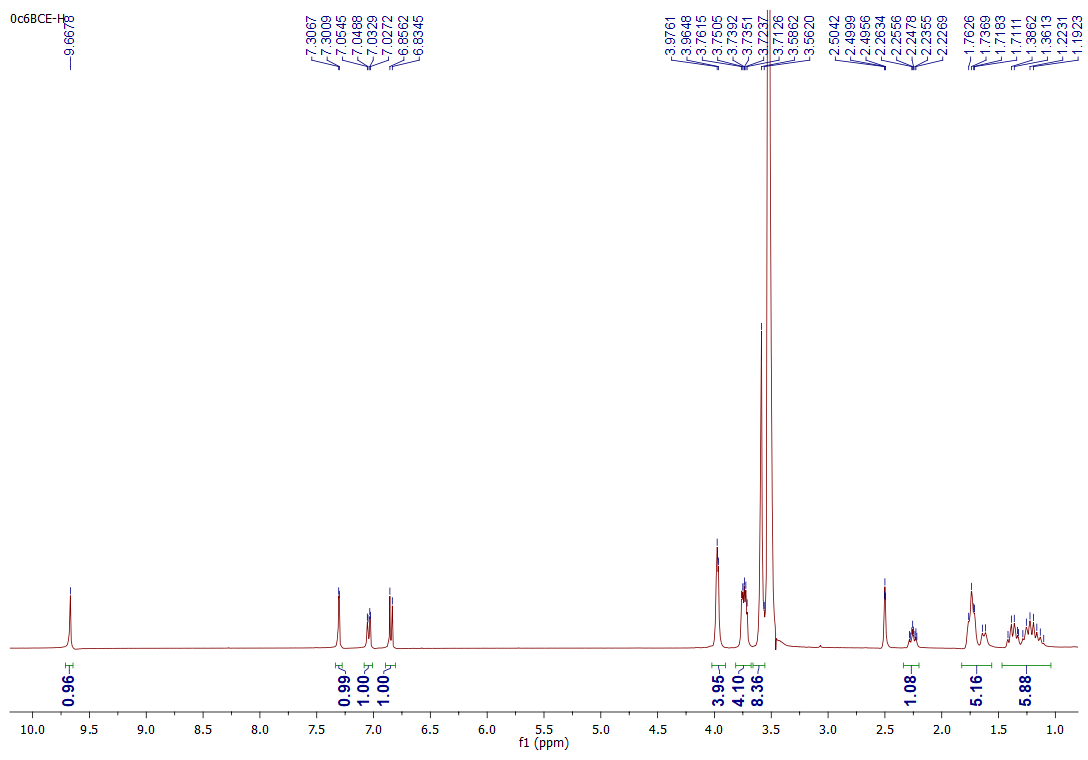


Compound **4**

Exact MW: **393.2151** g/mol

Exact MW: **393.2151** g/mol

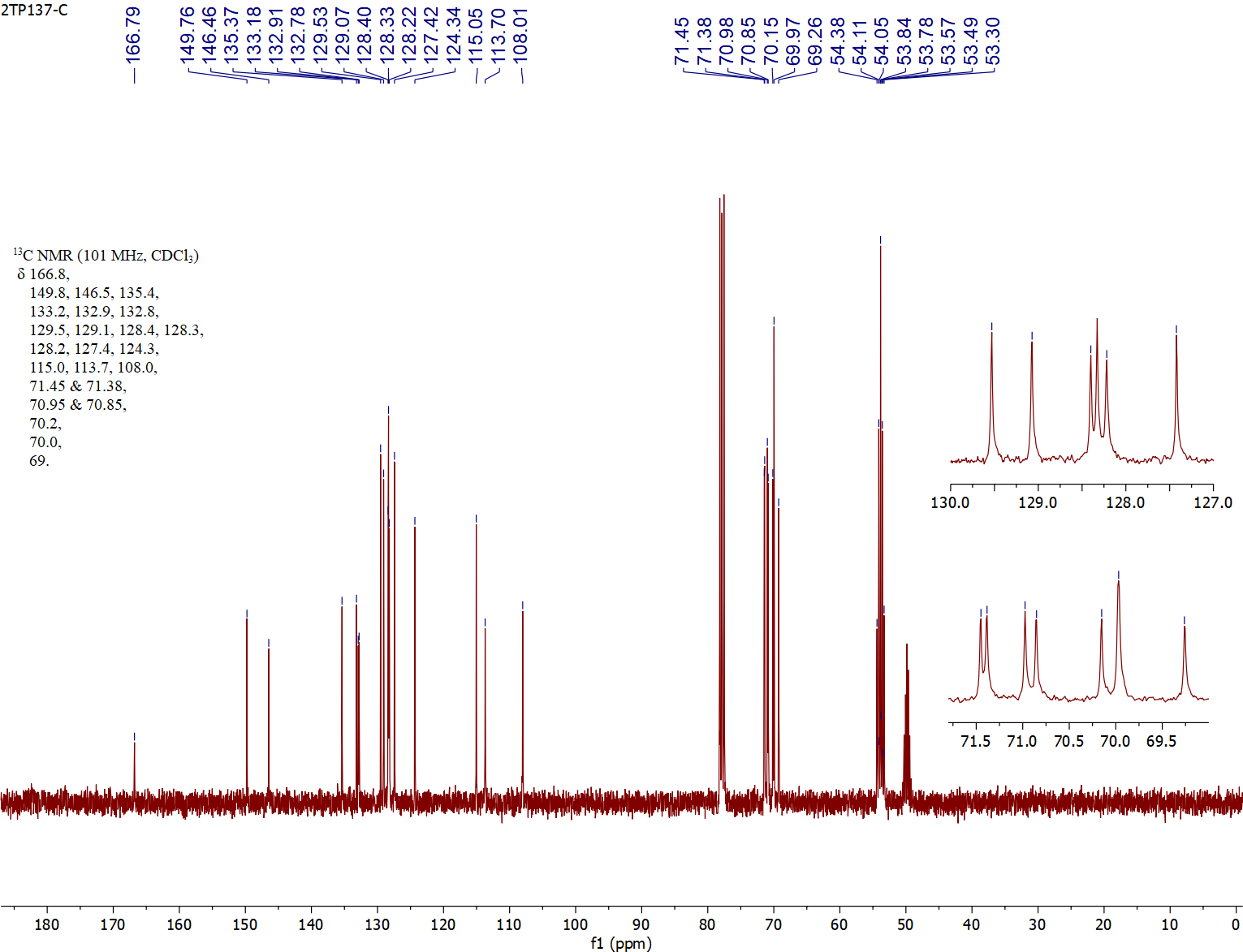


Compound **5**

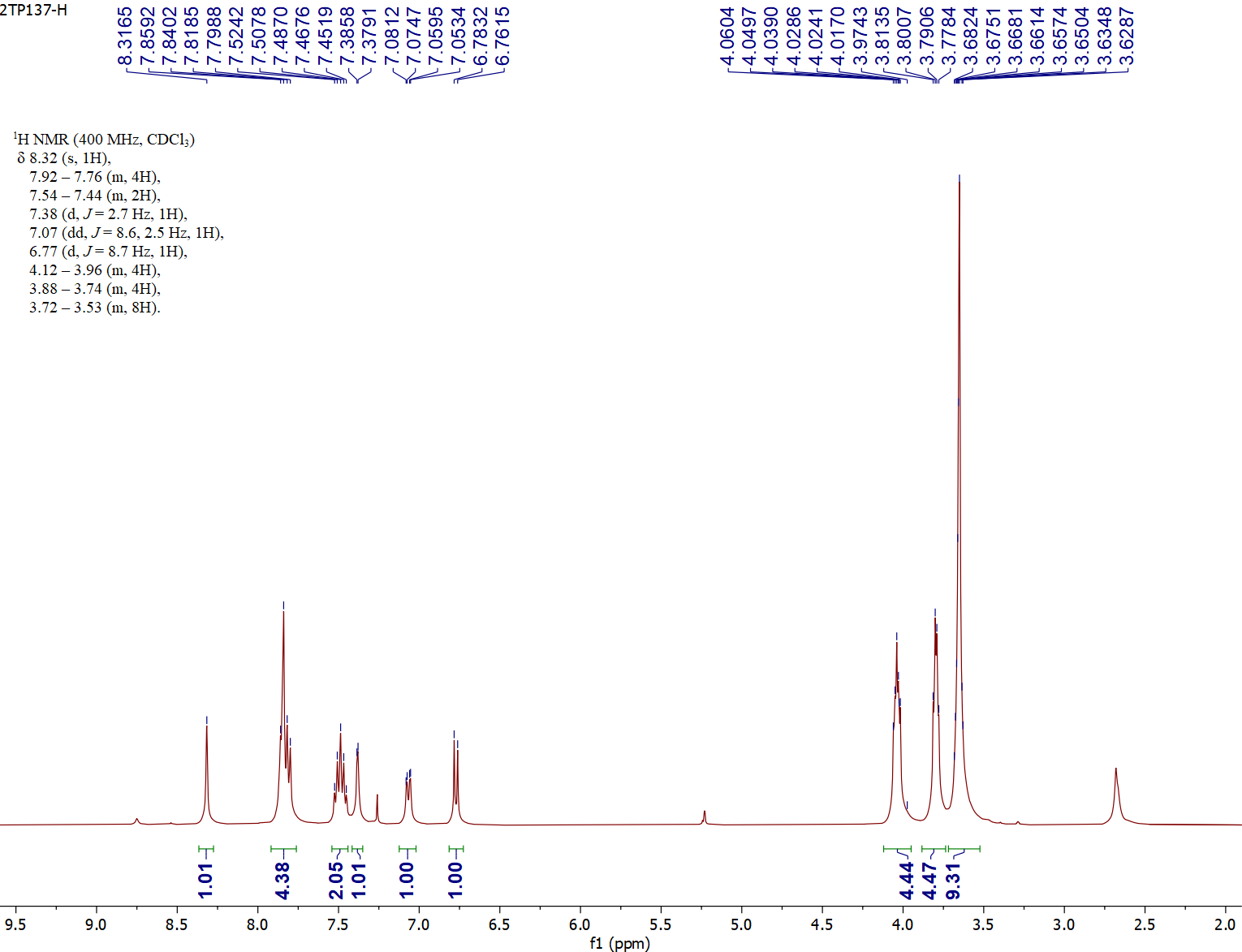


Compound **5**

Exact MW: **437.1838** g/mol

Exact MW: **437.1838** g/mol

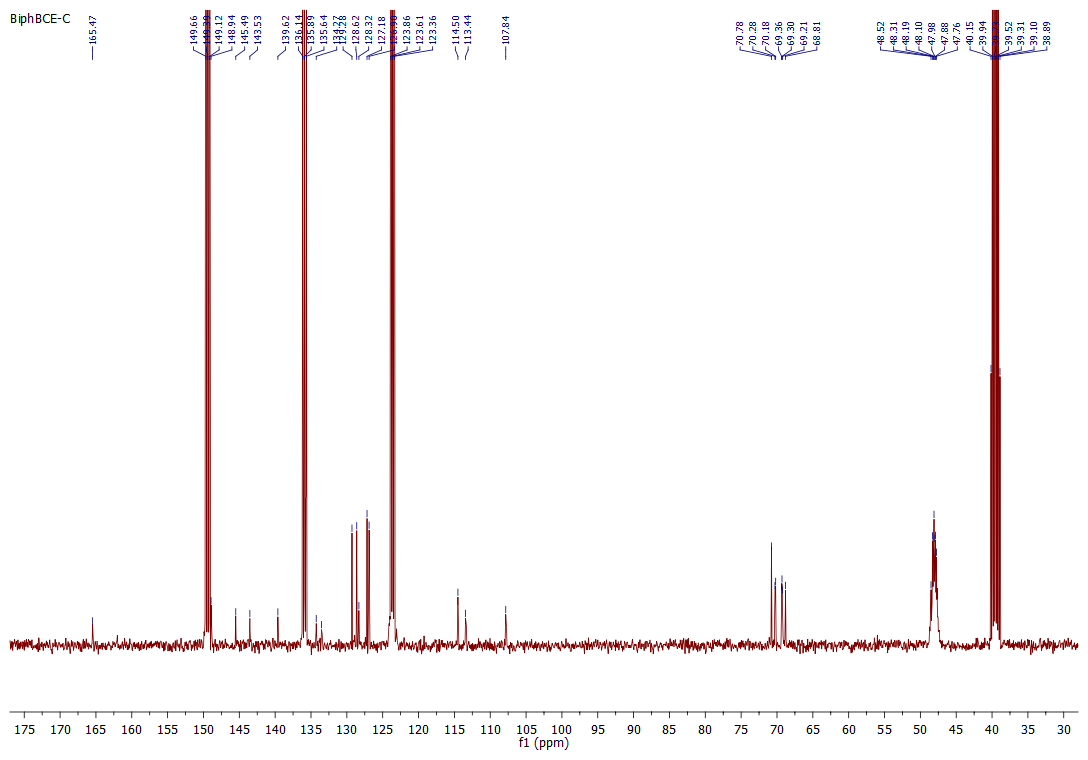


Compound **6**

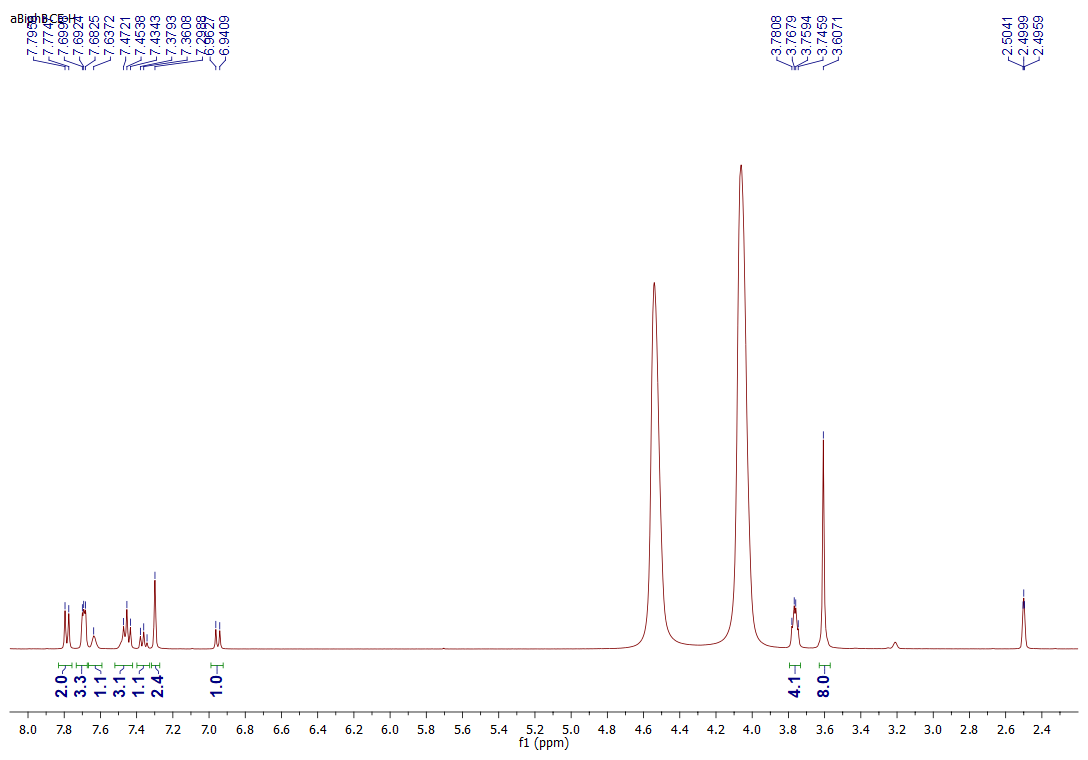


Compound **6**

Exact MW: **463.1995** g/mol

Exact MW: **463.1995** g/mol

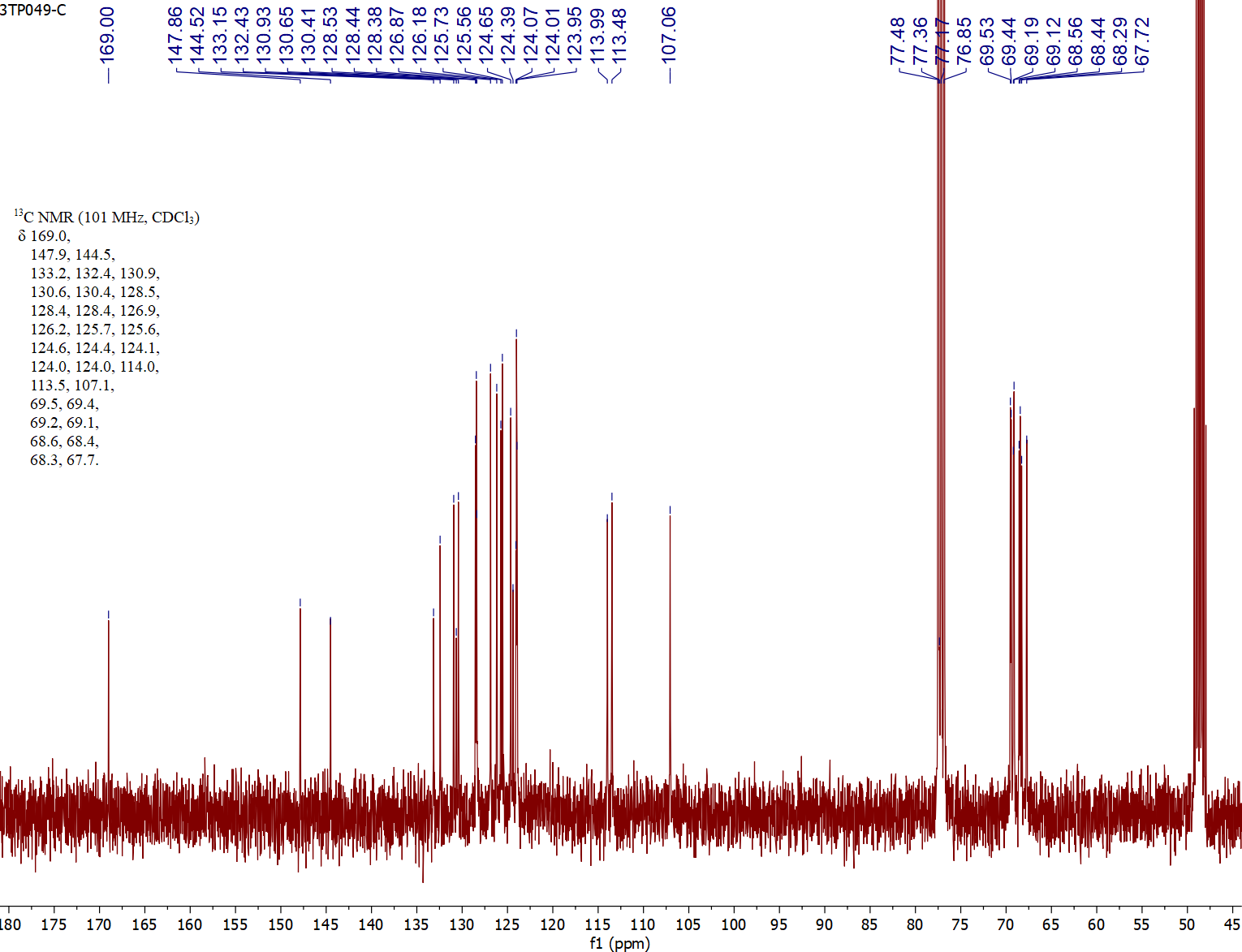


Compound **7**

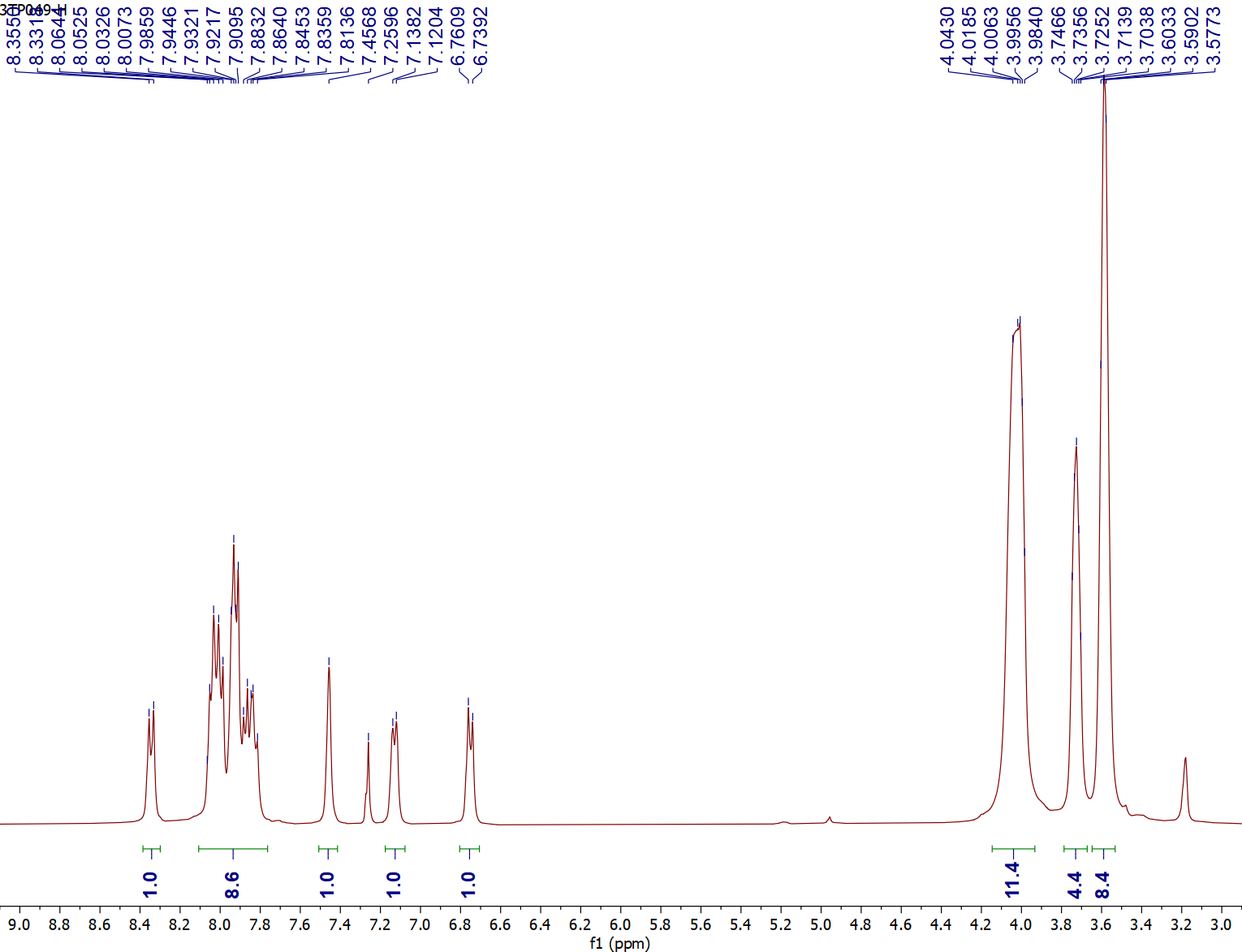


Compound **7**

Exact MW: **511.5740** g/mol

Exact MW: **511.5740** g/mol

# **References**

(1) Eadsforth, C. V.; Moser, P. Assessment of reverse-phase chromatographic methods for determining partition coefficients. *Chemosphere* **1983**, *12* (11), 1459-1475. DOI: <https://doi.org/10.1016/0045-6535(83)90076-0>.

(2) OECD. Test No. 117: Partition Coefficient (n-octanol/water), HPLC Method. *OECD Guidelines for the Testing of Chemicals, Section 1* **2022**.

(3) OECD. Test No. 107: Partition Coefficient (n-octanol/water): Shake Flask Method. *OECD Guidelines for the Testing of Chemicals, Section 1* **1995**.
